# Crop Biometric Maps: The Key to Prediction

**DOI:** 10.3390/s130912698

**Published:** 2013-09-23

**Authors:** Francisco Rovira-Más, Verónica Sáiz-Rubio

**Affiliations:** Agricultural Robotics Laboratory, Universidad Politécnica de Valencia, Camino de Vera s/n 3F, Valencia 46022, Spain; E-Mail: vesairu@upvnet.upv.es

**Keywords:** precision farming, global positioning, yield prediction, crop monitoring, vineyard management, precision viticulture, agricultural robotics, information technology

## Abstract

The sustainability of agricultural production in the twenty-first century, both in industrialized and developing countries, benefits from the integration of farm management with information technology such that individual plants, rows, or subfields may be endowed with a singular “identity.” This approach approximates the nature of agricultural processes to the engineering of industrial processes. In order to cope with the vast variability of nature and the uncertainties of agricultural production, the concept of *crop biometrics* is defined as the scientific analysis of agricultural observations confined to spaces of reduced dimensions and known position with the purpose of building prediction models. This article develops the idea of crop biometrics by setting its principles, discussing the selection and quantization of biometric traits, and analyzing the mathematical relationships among measured and predicted traits. Crop biometric maps were applied to the case of a wine-production vineyard, in which vegetation amount, relative altitude in the field, soil compaction, berry size, grape yield, juice pH, and grape sugar content were selected as biometric traits. The enological potential of grapes was assessed with a quality-index map defined as a combination of titratable acidity, sugar content, and must pH. Prediction models for yield and quality were developed for high and low resolution maps, showing the great potential of crop biometric maps as a strategic tool for vineyard growers as well as for crop managers in general, due to the wide versatility of the methodology proposed.

## 1. Introduction

Structural crises and widespread problems have historically been creative drivers of technology and innovative solutions, some of them ephemeral but very often inducers of philosophical transformations and even revolutionary outcomes. In 2006, millions of beehives worldwide emptied out as honeybees mysteriously disappeared, putting at risk nearly 100 crops that require pollination [1]. There may be no easy remedy to the colony collapse disorder; many suspects so far but no convictions yet, and solutions may require taking better care of the environment and making long-term changes to agricultural practices. This kind of structural changes in something as old as agriculture will likely require the advent of new technology in parallel with optimized data-based decision-making. Climate change, population growth, and increasingly scarce resources are putting agriculture under pressure [2]. Numerous North American specialty crops (fruits, vegetables, tree nuts, dried fruits, berries, and nursery crops), representing fifty percent of the total value of US crop production, are facing growing pressures that threaten their long-term viability [3]. Unfortunately, the implementation of technologies based on precision agriculture in practical farming has slowed in recent years on global scale compared to the mid- and late- 1990s [4]. In fact, until the late-1970s significant sums of money were invested in mechanization, robotics, and automation research and development in the US, but since that time, federal support to improve farm production through enhanced machine system has greatly declined, and therefore the research infrastructure for agricultural automation has deteriorated significantly over the past quarter century [3]. The reasons for this decay may rest in the difficulty to quantify benefits [5], the complexity of managing large amounts of data, and the intricacies of using advanced technology developed by academic or research institutions and hitting the market in an incomplete form [4].

In 2007, the US Department of Agriculture (USDA), the National Science Foundation (NSF), and the National Aeronautics and Space Administration (NASA), jointly sponsored a workshop to find the fundamental research and technology needs of specialty crops industries. Precision agriculture applications for yield *mapping*, yield and nutrient *prediction*, *data management*, *decision* support systems, and *diagnostic tools* run high among the key needs identified [3]. As a matter of fact, current farmers are fuelling a growing market for imaging systems where photonics is being used to gauge plant stress [2], and optics manufacturers confirm the move from descriptive techniques towards quantitative imaging, as machine vision facilitates objective measurements [6]. Even something as unconventional as space weather forecast for satellite-based applications may soon become common as precision agriculture practitioners recount how they depend on reliable access to high-accuracy global positioning [7]. Monitoring and mapping crops is, after all, like planetary explorations where truthful perception and accurate positioning must be efficiently synchronized, something that NASA's Curiosity rover achieves with no fewer than 17 cameras onboard.

Mapping for monitoring and decision-making necessarily involves sensing, measuring, processing, and real-time positioning. Crop inspection is largely done manually, but humans have a threshold beyond which they cannot see, and certain disease conditions are impossible to detect [2]. Thermal imaging, for example, has been successfully used to monitor tree canopy in citrus, providing a record of the temporal variation of vegetation that allowed the detection of the fruits, and therefore an estimation of yield, although the lack of geographical references prevented the general assemblage of maps [8]. Gauging crop yield months before the harvest is not easy, thanks to a host of elements that can impact growth and often are out of a farmer's hands [2]. A reliable and low-cost method of generating yield maps of citrus has been by localizing hand-harvested containers of oranges with a GPS recorder, acknowledging yield variations within a citrus block, and allowing surface interpolation of yield data. This straightforward technique is applicable to other crops with little or no modification, although it does not map the yield of individual trees as desired by many growers [9]. In addition to yield, there are many other parameters to monitor before harvesting. Field-based, high-throughput phenotyping seeks to implement information technologies to characterize the growth response of genetically diverse plant populations in the field, which practically hinges on the availability of a proximal sensing system [10]. In fact, although remote sensing pioneered many applications of precision farming —especially related to hyperspectral vision–, resolution, grower controllability, and the need of high update rates practically unbalance the scale in favor of proximal sensing. Citrus groves, for instance, are aerially photographed in Florida at least once every two years for taxation purposes [9], which obviously is insufficient to monitor crop parameters along the season. Being Florida citrus one of the most technology-driven crops in the World, other regions will certainly have a much lower update rate, and thus remote sensing cannot offer the degree of flexibility required by most of medium and small growers in a global scale. Yet, satellite imagery may result helpful to validate ground data, as the weed mapping system developed to measure weed intensity and distribution in a cotton field [11]. Ground measurements were carried out with the Weedseeker sensor module in combination with a GPS, and later compared to remotely sensed imagery in order to predict crop canopy coverage, which eventually was most closely correlated with the Normalized Difference Vegetation Index (NDVI) plus weed intensity at a coefficient of variation 0.2 ≤ *R*^2^ ≤ 0.53. The majority of the estimations, measurements, and predictions made before harvesting are oriented to enhance mechanized or robotized harvesting, where expert systems somehow try to emulate and substitute the skills of pickers. The European Commission-funded DASH project has developed a working prototype of an asparagus-picking robot, currently being readied for market, and imaging systems are being introduced in Europe to sort grapes according to the quality of the wine they will produce [2]. Once sorted, the grapes may be collected by an autonomous machine such as the Japanese multipurpose robot capable of harvesting, berry thinning, spraying, and bagging of the grape bunches [12].

The main objective of the research reported in this article is the establishment of a framework to take the rich ideas and concepts behind precision agriculture and information technology to the reality of orchards, proposing a procedure for handling large amounts of data generated by advanced systems but targeted to users with no high-tech education. A step-by-step application of this methodology to such a high-value crop as wine-making vineyards illustrates the key stages of the method and demonstrates the real potential of crop biometric maps.

## 2. Conceptual Foundation of Crop Biometrics (CB)

The economic and social reality found in industrialized countries, where production costs in agriculture keep growing while produce maintain ever-decreasing prizes, benefits from the efficient application of information technologies (IT) to agricultural production, in such a way that specific information at plant, row, or subplot scale can be attained. This idea approximates the nature of agricultural processes to the mechanics of industrial processes in what could be perceived as a *naturalization* of control system. However, agricultural production processes are further challenged by an enormous variability and the uncertainties of working outdoors in uncontrolled environments. The scientific and systematic study of agricultural processes is instrumental to increase the quality of products, enhance management efficiency, and develop prediction models. Prediction, in particular, is crucial for the right management of many crops. With the purpose of setting the ground for prediction in agricultural production, the term *crop biometrics* is defined as the *scientific analysis of field observations confined to spaces of reduced dimensions and known time-invariant position*. The practical realization of this idea involves deciding which physiological –or biometric– identifiers must be selected for each application, the optimal size of each individual space holding the magnitude of a biometric trait, the way to quantize traits and predicted parameters related to yield and quality, the mathematical or statistical relationship among traits and predictions, and the scope of predictive models based upon the biometry of specific crops.

The idea of crop biometrics is not far away from the concept of human biometrics, from which it gets the inspiration. For the human case, it can be defined as the automated recognition of people *via* distinctive anatomical and behavioral traits [13]. Nevertheless, although both terms focus on biological traits, and therefore both need to make decisions on the basis of imperfect measures, the operational philosophy is remarkably distinct. The most significant differences are the following: first, the purpose of crop biometrics is predicting the outcomes of agricultural processes, assuming that these models make predictions according to imperfect measurements; secondly, unlike human biometric traits, crop traits are not unique, rather, it will be the opposite as many plants of the same field will share similar or identical vegetative vigor, production yield, or quality indices; thirdly, plant biometric traits change with time over the season, which is just the opposite to the immutability of, say, fingerprints; fourthly, sensors for crop biometrics are not always low cost; and finally, the holistic concept of crop biometrics includes factors that affect the plant but are not a part of it, as water availability, sun radiation, or soil resistance found by the roots, yet all can be enclosed in the same working site and be statistically related.

The practical implementation of the concept of crop biometrics requires making important technical decisions:

1. The selection of specific *crop biometric traits* (CB-traits) depending on each particular application, crop, or managerial need. A tabular format for the appropriateness of potential traits may be helpful at this stage of the process. The definite set of traits will always remain opened to new additions or the removal of poor performance traits. Table 1 provides an example of potential CB-traits for vineyards.
2. The establishment of a protocol for the measurement of traits, specifying the procedure, the sensors, and the time and frequency of the estimations. Such issues as the sensitivity of the measurements in relation to the size of the cells must be addressed along the process.
3. The design of the grid, determining mesh resolution and cell size.
4. The method for analyzing the traits, verifying their statistical significance and establishing correlations among traits to propose prediction models with a known level of uncertainty.

**Figure 1.**
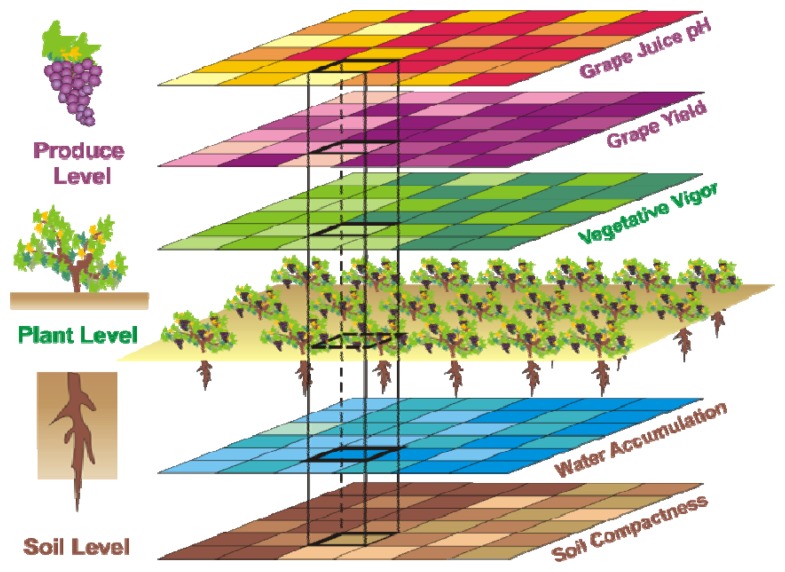
Tri-level division of traits for vineyard management.

The vineyard case enounced in Table 1 will be further developed to validate the idea of crop biometrics. It features a tri-level division of traits given by *soil* level, *plant* level, and *produce* level traits. Figure 1 schematically shows the multi-level compatibility of maps that is necessary to establish prediction models. Table 1 lists some of the crop traits of interest for the vineyard application. However, not all of them ended up being helpful, and by contrast, future traits not considered here will probably play a key role in the definition of future models. Alternative parameters such as soil conductivity, nitrogen content in leaf, sun radiation, leaf temperature, canopy density, phenolic status of grapes, or even laser-based carbon dioxide absorbed and emitted by foliage in the photosynthesis [14] might be instrumental for the efficient management of the vineyard of the future.

## 3. Selection of CB-Traits

The goal behind the idea of crop biometrics is to provide an IT-based management tool for modern agriculture based on two core principles: the construction of compatible user-friendly crop maps, and the representation of key information for the grower through CB-traits. As a result, in order to build useful maps, CB-traits must be carefully chosen according to particular field needs. However, in addition to their interest for the grower, there exist other important factors that need to be taken into account too, as the list of properties considered in Table 1. The ideal situation occurs when a trait is essential for the grower, it can be measured automatically, quickly, at low cost, and is well correlated with the parameters being predicted, usually yield and quality. For the particular case of wine grapes, the quality of the future wine is even more interesting than the quality of grapes at harvesting time, and consequently, the predictive nature behind the concept of crop biometrics results in a strategic tool for wine makers. Unfortunately, the majority of CB-traits do not comply with these ideal properties, but this should not be a cause for rejection; rather, any CB-trait that adds value to the solution must be considered, even if the sensors currently available are overprized or difficult to automate. Forthcoming research will eventually palliate these inconveniences and by the time unaffordable sensors become accessible, the already existing framework to process their data will result in higher accuracy for the models and smoother integration for the sensors. This could be the case, for example, of the assessment of soil compaction and the measurement of grape juice acidity in Table 1; thus far, both traits are manually sampled, but future scouting robots may be capable of conducting sampling missions autonomously, increasing the amount of data while reducing time and cost. Whenever this becomes available, the elaboration of these particular maps will be faster and better, but the procedure to integrate data in the predictive models will be exactly the same followed with the manually-generated maps, as all maps –new and old– are designed to be compatible among them and with the rest of the maps included in the model. As a matter of fact, the development of automated measuring systems is in continuous expansion, with new solutions for soil sampling and phenolic maturity reaching real time performance.

As shown in Table 1, properties of different nature must be confronted to candidate CB-traits before choosing the set of traits associated to a given application, as what is interesting for a crop may not be appropriate for others. Vegetative vigor, for example, is known to influence grape yield and wine quality, but it will probably result in a poor indicator to predict yield in an orange grove. As a result, each particular application requires a customized CB-traits table. In the construction process of a trait-property cross table, the following points should be considered:

1. Cost induced by the trait, including the prize of purchasing the sensor plus the expenditures involved in the measuring process.
2. The more automated a measurement is, the lower reliability it tends to have.
3. The interest of farmers in tracking certain traits, as it varies with applications and may differ for the same crop cultivated in diverse locations.
4. The strength of correlation among CB-traits establishes the validity of predictive models, and typically requires the support of statistics. In that respect, reliability in the measurements must be assured before applying statistical methods of analysis. Even so, statistical procedures are not intended to replace subject-matter judgments based on theoretical knowledge and field experience.

## 4. Measurement and Positioning of CB-traits: Map Construction

The methodology to process and handle biometric information is as important, or even more, than the actual acquisition of data. Some sensors can provide a precise measurement in less than a second but the area per sample is large. Other times, instead of a sampling probe, crop information is gathered from digital images. Yield monitors are designed to estimate instantaneous yield on-the-go. How can all this information be efficiently combined in a standard map? Two principles account for the management of crop biometric information with compatible maps:

1. The selection of a convenient coordinate system with functional axes guarantees repeatability during a season and compatibility over the years. In addition to this, Euclidean geometry facilitates the measurement of distances and the calculation of areas, especially if compared to spherical (geodetic) coordinates. All these conditions are met by the Local Tangent Plane (LTP) coordinate system, as it uses the Cartesian axes north, east, and altitude, and allows the selection of a local origin chosen by the user, and set for all the crop maps associated to the field analyzed.
2. The homogenization of data through regular grids of user-selected resolution, regardless of the nature of the sensor implemented, its sampling rate, or the area covered per measurement. Given that the origin of coordinates in each field can be fixed by the producer, and the size of the grid's cell is kept constant through time, the resulting crop maps can be easily standardized for each given field, resulting in a grid format of determined resolution. This procedure leads to important implications, as biometric information and future predictions for a field should be freely exchanged over time and space. Local origins and intuitive coordinates help farmers and field managers relate map cells with the actual terrain. Even if the resolution of the grid is changed by modifying the cell size, crop maps can still be compared zone by zone, and therefore compatibility is always granted. Cells without information do not create any problem because the global positioning of cells allows the completion of maps in subsequent passes and data correlation only occurs among cells storing biometric data.

The fulfillment of these two principles allows the comparison and correlation of compatible maps carrying biometric information. However, several subtleties need to be further discussed before assembling the set of crop maps that characterize a field. To begin with, the relationship between cell size and the nature of the CB-traits should be investigated in detail. Generally speaking, there will be many traits but only one cell size will be adopted for all the maps. Obviously, the measuring technique of each trait sets the smallest size under which additional subdivisions are meaningless. For example, if soil is sampled every 5 m along a row separated from its neighboring rows by 6 m, square cells of 3 m size will result in many empty cells, but expanding the cell size to 6 m, 10 m, or 15 m will lead to alternative maps representing equivalent biometric information at different resolution. When several measurements fall inside the same cell, the magnitude of the traits is averaged to provide the mean value of the trait corresponding to that cell. Naturally, the bigger the cell the less accuracy will have the model, as specific crop information is lost through the averaging process. Some applications, however, may require labeling the cells with the top values rather than the averages. In any case, map variability will increase as cell size diminishes. As a result, the biometric parameters represented in a map possess certain sensitivity to the actual size of the cells (map resolution), mainly given by the equilibrium between sampling rate and sampling spatial range. Therefore, the right trade-off must be established in such a way that the information carried by each particular cell is meaningful by itself and in relation with the rest of the crop map.

This section provides the framework to build crop biometric maps in general terms, but the specific equations and detailed algorithms to assemble them fall outside the scope of this paper. The following references may help to apply these ideas to particular cases. The transformation from geodetic coordinates to the local tangent plane is explained step-by-step in ([15], Chapter 3, pp. 68–71). The construction of regular grids with global references given in the LTP coordinate system is described in [16]. The implementation of conditioning filters to enhance the robustness of GPS data can be checked in [17], and finally, the estimation of spatial variation of vine vegetation with machine vision has been reported in [18]. Section 6 applies this methodology to the particular case of vineyards, presenting more insights and practical solutions on the proposed philosophy.

## 5. Mathematical Analysis of CB-Traits

In the methodology proposed, the working unit is the cell of a CB map, and consequently everything happens at cell level. This implies that there will be a set Z of *n* biometric traits Z = {T_1_, T_2_, …, T_n_} representing diverse crop-related properties, and a set of cells forming a map where the elements of Z are represented, so that the total number of maps related to a field will be greater or equal than *n*. As a result, CB maps may be correlated at cell level–*i.e.*, cell by cell for equivalent positions– and checked for statistical significance among traits in such a way that prediction models may be enounced for a certain subset of Z. Predicted traits must be eventually evaluated according to their proximity to the actual measurements determined by the “ground-truth” verification conducted in the field under study. Again, this evaluation must take place at cell level. Every CB map will have a horizontal resolution of *h* cells and a vertical resolution of *v* cells, summing up a total of *h*·*v* cells. Map cells can be identified using the standard matrix notation T_k_ (*i*, *j*) where *i* = {1, 2, …, *v*}, *j* = {1, 2, …, *h*}, and *k* = {1, 2, …, *n*}.

The predictive models inferred from correlating a selected number of traits will always be statistical models rather than mathematical models, as they cannot represent precise relationships free of error but approximate relations deduced from data prone to experimental errors. The statistical analysis of CB-traits proceeds according to the following actuation protocol.

### 5.1. Statistical Nature of Selected CB-Traits

Before making any attempt of establishing statistical correlations among different traits, it is important to analyze the statistical nature of the selected traits. In particular, if they represent stochastic (or random) variables and how they behave in terms of basic statistics. These properties are key to explain variability within the field, the foundational concept behind precision agriculture. A random variable usually takes on a set of possible values, each with an associated probability, which conceptually may represent the subjective randomness (crop variability) resulting from incomplete knowledge on the biological processes behind crop production. From that standpoint, crop traits can be considered random variables, even though their values are not intrinsically random, because measurement errors tend to follow a random distribution. In fact, normally distributed errors are assumed for regression models, F-tests, and ANOVA [19]; therefore, the *assumption of normality* should be checked for the set of traits proposed in the study of a field, and if data behaves approximately normal, the set of conventional statistical tools can be used to generate predictive models. The normal quantile-quantile plot provides a direct evaluation of the assumption of normality, where approximate linearity indicates normally distributed errors.

Once the assumption of normality has been verified, inferences on means and standard deviations may be properly interpreted. At this point, especial attention must be paid to the appearance of *outliers*, *i.e.*, extreme values with respect to other observations made under the same conditions. When sensors and other electronic devices are set to gather data for long periods of time under tough environmental conditions, noise is prone to appear, and predictive models based on regression may result extremely affected by uncontrolled outliers. Therefore, provisions should be made to deal with unrealistic data before composing the CB maps. In general, two approaches can be followed with regards to outliers: identification, determining what observations are outliers for their removal; and accommodation, mitigating their effects within the map. If the presence of outliers becomes an operative problem, the technique of studentized deleted residuals can be used for outlier-detection statistics.

### 5.2. Coherence between Equivalent or Related Biometric Traits

As electronic and information technologies rapidly evolve, more and new measurement techniques for crop traits will become available. Under these circumstances, it will not be uncommon to end up collecting different maps of the same or closely related traits, as the alternative vegetation coverage estimated in Section 6 from digital images taken with two different fields of view. As several CB maps model the behavior of a unique trait, the information reported, while different, must be equivalent. This fact must be verified by establishing correlation models among equivalent quantifications of the same trait. In fact, predictive models will mostly need the participation of only one type of measurement per significant trait, and thus the best correlated parameters should be identified before determining the definitive predictor variables of the model.

### 5.3. Enunciation of CB Prediction Models

A CB prediction model is the regression-based estimation of a quantitative variable related to produce yield or quality given as a function of one or several predictor crop traits, establishing a statistical correlation among traits with a solid foundation for dealing with observational data. However, when analyzing data in which most of the variables are not controlled, extreme care must be taken to ensure that proper inferences are drawn when statistically significant results are obtained [19]. *Confounding* is especially important as the effect of traits may not be uniquely ascribed to the subset of traits considered as predictor variables. Since the evolution of traits in the field runs mostly uncontrolled, confounding is likely to occur, and modesty should always be present in the claims made about predictive models.

The regression models deduced for making CB predictions are mathematically based on least-squares estimates, and initially may be linear, multivariate, or polynomial. For least-squares predictions of the simplest linear type, the slope is related to the Pearson's product-moment correlation coefficient *r* in such a way that both are equal when the standard deviation of both variables (predictor and predicted) is the same [19]. In such case the absolute value of the slope necessarily has to be less or equal to one because |*r*| ≤ 1. As a result, slope values under 1 cannot be interpreted as a lower response because this effect is expected if the variability of involved variables is similar. Consequently, the standard deviation of all traits employed to compose CB maps must be carefully examined before drawing conclusions on linear models. For traits having analogous variability, not rare if the same trait has been studied through alternative measurements as mentioned above in Section 5.2, this possibility, formally known as the *regression fallacy*, needs to be verified. Nevertheless, predictions will generally require the combination of various crop traits, and multiple linear regression provides an upgrade of linear regression where the magnitudes of least-squares coefficients measure the change in the prediction due to a unit change in one trait while all remaining predictor traits are held constant. However, this is not always so straightforward due to potential interrelationships among traits which may result in drastic changes in predictions when a trait is added or deleted. In fact, given that traits are typically measured on different units, it is inappropriate to compare the model's coefficients directly. As a general rule, a predictive model should not routinely insert products of all the traits initially considered as significant, as doing so will likely create unnecessary complexities in the analysis and interpretation of the predicted trait due to *collinearities* among predictor traits. Crop biometric models are not known before the analysis of the data. The underlying mechanisms that correlate traits are not well understood due to the inherent complexity of the problem and the lack of sufficient theory. This lack of deterministic information opens the solution to any kind of functional relationship, being polynomials and logarithms good candidates in which satisfactory approximations can be found. The final selection of the model type, however, must be done with prudence, starting with the simplest model suggested by scatter plots of the traits and the biological mechanism under study. In that respect, linear models will usually comprise the initial steps, moving to nonlinear models when there exists a clear advantage. In either case, the region of prediction must be well defined such that extrapolation never occurs, as the validity of the predictive model cannot be guaranteed outside the working interval.

The measure of goodness of a fit is crucial to select a CB prediction model. Several indicators may be helpful to make an educated decision on which model will yield the most reliable prediction for the established working interval. In particular, the following checks may contribute to add relevant insights to the evaluation of candidate models: F-statistics and p-values from ANOVA tables; analysis of t-statistics for assessing collinearity effects among traits; empirical judgment and theoretical considerations on the crop; stepwise selection of traits, either adding or backward suppressing; and calculation of the sample Pearson's correlation coefficient *r* or the coefficient of determination *R^2^*. Overall, caution should be used in relying on a single measure of the fit, such as *r* and *R^2^* values. As a matter of fact, there is an extended tendency to over rely on *R^2^* (= *r^2^*) because of its straightforward interpretation. To begin with, the use of Pearson's *r* is appropriate only when the variates are stochastic variables. It can be calculated when some of the variables are not random but the calculated value of *r* is simply a measure of the degree of least-squares balance between the error sum of squares and the total sum of squares. Kvålseth [20] affirms that the coefficient of determination *R^2^* is widely misused, and special care should be taken when comparing fits between models with and without an intercept term, linear and nonlinear regression models, and models in which the response variable is not in exactly the same functional form. Out of the eight alternative expressions of *R^2^* analyzed by Kvålseth [20], the recommended choice of *R^2^* statistic for linear models with or without intercepts, for nonlinear models that are intrinsically linear, when linear least squares regression is used, and even for models that are intrinsically nonlinear and fitted by nonlinear methods is shown in Equation (1), where *y_i_* are the field measurements of the trait T_k_ that is being predicted with the model, *ŷ_i_* denotes the predicted values for trait T_k_, and *ȳ* denotes the arithmetic mean of *y_i_*. The potential limitation of Equation (1) to assess the goodness of predictive models is the lack of resistance to extreme values, as this calculation of *R^2^* has a relatively low degree of resistance to outliers. At the high variability and dispersion of field measurements, it would be desirable to use an *R^2^* statistic with certain resistance to marginal estimations. Such a statistic $R_{\text{res}}^{2}$ may be derived by simply replacing the arithmetic means of Equation (1) by sample medians, as illustrated in Equation (2) [20]:

(1) $$R^{2}=1-\frac{\sum {\left(y_{i}-{\hat{y}}_{i}\right)}^{2}}{\sum {\left(y_{i}-\bar{y}\right)}^{2}}$$

(2) $$R_{\text{res}}^{2}=1-{\left(\frac{\text{Median}\{|y_{i}-{\hat{y}}_{i}|\}}{\text{Median}\{|y_{i}-\bar{y}|\}}\right)}^{2}$$

## 6. Results and Discussion: Vineyard Biometrics

The main advantage of *crop biometric maps* is their practical applicability to any kind of crop and any size of field. The general principles of this methodology have been described above, but the specific adaptation to a particular case requires the selection of predicted traits, predictor traits, grid resolution, and local origin of coordinates. In addition, right after choosing the principal traits to be monitored on a crop, a thorough description of the procedure to measure the traits must follow. The particularities of trait measurements will eventually suggest the most appropriate cell size, and hence the grid resolution for all CB maps. This section provides a detailed study case where the CB methodology has been applied to a wine-producing vineyard with the purpose of predicting grape yield and the site-specific quality of the future wine.

### 6.1. Construction of Vineyard Biometric Maps

**Figure 2.**
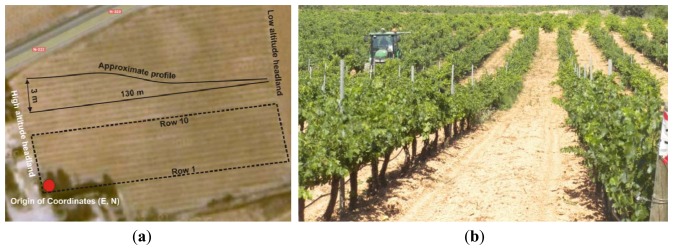
(**a**) Top view of experimental field: row selection; (**b**) Vine status at testing period between *véraison* and grape harvesting.

The crop biometric maps envisioned to establish a predictive framework on yield and quality were developed for the ten rows of grapevines highlighted in the top view of Figure 2a. The field is located in Requena (Valencia, Spain), and consists of 20-year-old Cabernet-Sauvignon vines along trellised rows spaced 3 m and 130 m long with a 3% average slope, as shown in Figure 2. The approximate placement of the local origin of coordinates is marked in Figure 2a by a dot, set at the highest elevation point of row 1 from where the image of Figure 2b was taken. The cell size established for all the crop maps was 4 m (square cells) leading to a working area unit of 16 m^2^. The following traits were initially selected to construct predictive models for grape yield and quality:

1. Average soil resistance to root growth estimated by 218 standard penetration tests.
2. Maximum soil resistance to root growth estimated by 218 standard penetration tests.
3. Water content in soil indirectly estimated by partial elevation above lowest field point.
4. Relative vegetative vigor assessed with images automatically taken with an 8 mm lens.
5. Relative vegetative vigor assessed with images automatically taken with a 12 mm lens.
6. Grape yield manually weighted at 219 sampling points in the field.
7. Sugar content measured in degrees Baumé from 219 grape samples.
8. Must acidity.
9. Must pH.
10. Weight of 10 berries.
11. Average diameter of berries.
12. Average berry density.

**Figure 3.**
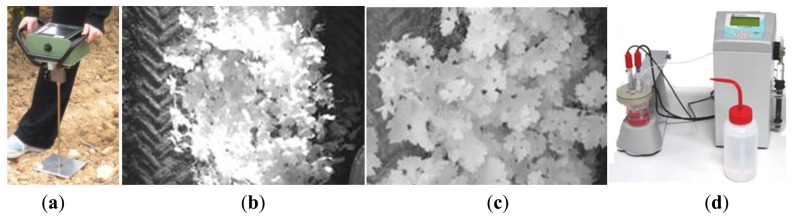
(**a**) Measurement of soil compactness; (**b**) Relative vegetation images with the 8 mm lens; (**c**) Relative vegetation images with the 12 mm lens; (**d**) Acidity and pH measurements.

The soil resistance to root growth, both average and maximum, was measured in MPa by standard penetration tests conducted with the handheld penetrometer of Figure 3a (Eijkelkamp Agrisearch Equipment, Giesbeek, The Netherlands). Global references for the sampled points were acquired with a portable GPS antenna-receiver (Leica Geosystems, Heerbrugg, Switzerland). The vineyard is planted in sloping terrain, and although irrigation can be controlled by the producer, water tends to accumulate in the lowest section of the field at the eastern side. The content of water in the soil was assumed to be inversely proportional to the relative elevation of the sampled cell over the lowest headland in the field (marked in Figure 2a). Elevation data was automatically registered in cm by a tractor equipped with a GPS receiver (StarFire iTC, Deere & Co, Moline, IL, USA) providing NMEA messages at 5 Hz and conditioned by the algorithms of [17]. The spatial variability of vegetation was quantified from zenithal images taken with a monochrome camera (JAI CM-140GE-UV, Copenhagen, Denmark) centered in the near infrared (NIR) band. Two assessments of vegetation were conducted according to the field of view of the camera: wide view with 8 mm focal length (Figure 3b); and narrow view with 12 mm focal length (Figure 3c). NIR-filtered images enhance vegetation from the background, and a customized dynamic segmentation algorithm [18] was developed to quantize the variation of the amount of leaves along the rows. The camera was mounted on a side bar attached to the tractor's cabin, and the vehicle's GPS provided its instantaneous position associated to each image automatically taken, following the architectural principles set in [21]. Relative vegetation was expressed in percentage, representing the number of pixels belonging to vegetation from the total number of pixels in the images of resolution 696 × 520. According to this definition, higher values of relative vegetation are expected for the images taken with the 12 mm lens, as the background representing soil or vehicle parts will be less apparent in the narrow-view images. The assessment of grape yield is crucial for this application, as it is a trait that involves predictions as well as measurements. The yield map was constructed from weighting the grapes harvested along the rows with a digital dynamometer (Mecmesin, West Sussex, UK) for each interval of 4–5 m, totaling 219 measurements evenly distributed over the 10 rows mapped. The coordinates of the center points of the virtual areas from which grapes were manually harvested were recorded with the hand-held portable GPS. When the grid was formed, those cells including a yield measurement were labeled with the corresponding yield measured in kg; cells with more than one sample averaged the yield measurements, and empty cells remained with no data. At the time yield was being estimated by intervals, a representative sample containing various grape clusters was extracted from each interval and taken to the laboratory for its further analysis. In the laboratory, the sugar content of the must was measured in degrees Brix (Balling) with a digital refractometer (DR101Comecta S.A., Barcelona, Spain), the total acidity and pH of the must was measured with the semi-automatic tritator and ph-meter of Figure 3d (PH-Burette 24 Crison Instruments S.A., Alella, Spain). Berry weight was measured with a precision scale and the average diameter of the grapes was estimated with a digital caliper (Harbor Freight Tools, Camarillo, CA, USA).

**Figure 4.**
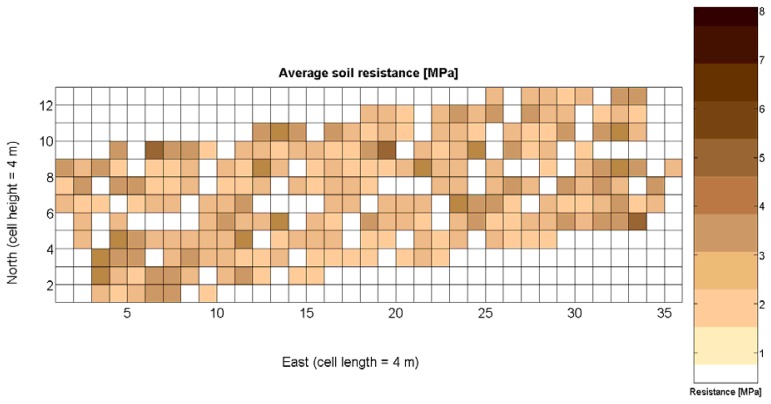
CB map of average soil resistance (MPa).

**Figure 5.**
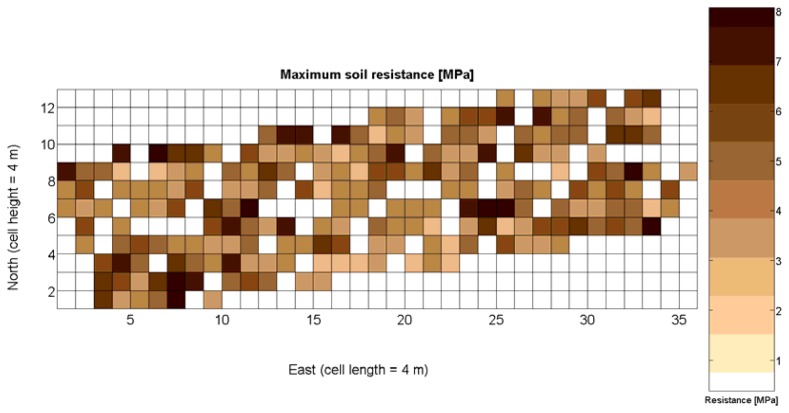
CB map of maximum soil resistance (MPa).

**Figure 6.**
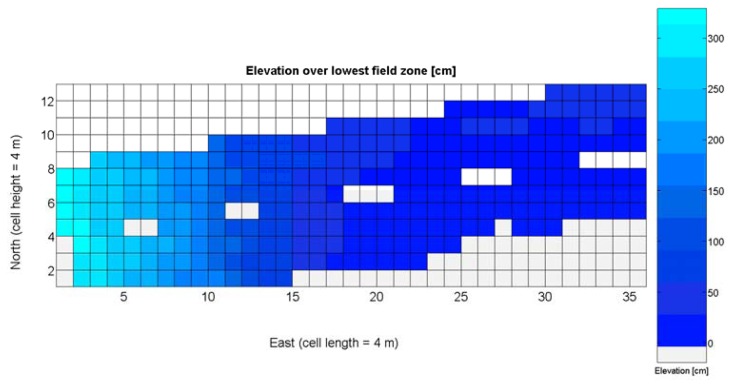
CB map of field relative elevation (cm).

**Figure 7.**
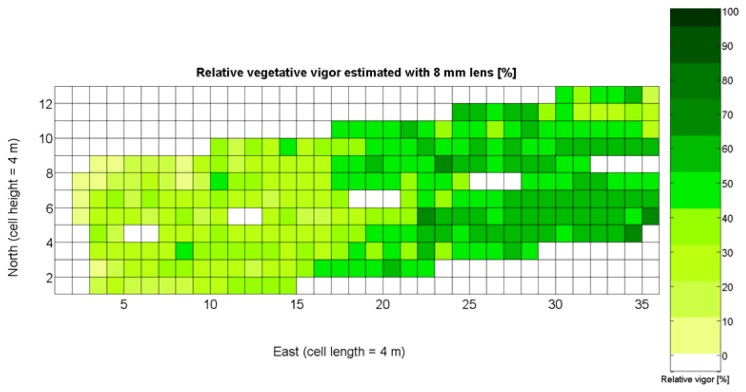
CB map of relative vine vigor estimated with an 8 mm lens: V-8 (%).

**Figure 8.**
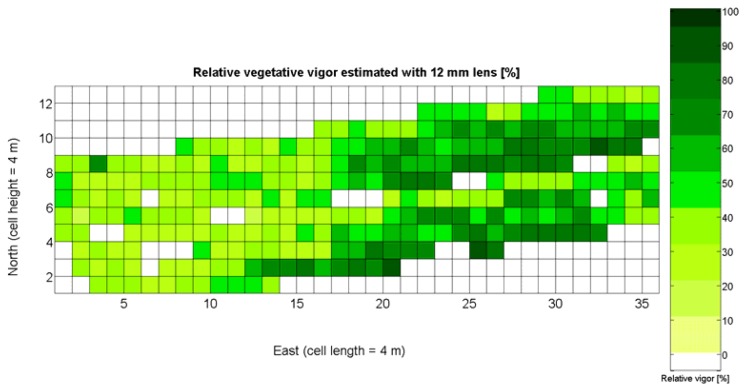
CB map of relative vine vigor estimated with a 12 mm lens: V-12 (%).

The 12 traits listed above were represented in compatible CB maps, where every cell is uniquely identified and physically related to an area in the field, as conceptually represented in Figure 1. As a result, crop traits may be compared cell-wise as long as there is information available for the chosen cells. A given cell may contain biometric information relative to certain traits out of the 12 initially considered; therefore, statistical correlation will be available among data-carrying cells. Figure 4 represents the CB map for the average soil resistance in MPa whereas Figure 5 provides the maximum soil resistance. Notice that both maps share the same resistance scale, and comparisons are possible in a cell to cell basis. Figure 6 is the elevation map of the ten rows analyzed in the field, and permits an indirect assessment of the water stored in the soil throughout the season, being lower elevation cells (mapped in dark blue) an indicator of higher moisture in the soil. Figures 7 and 8 map the spatial variation of vegetation between *véraison* and harvesting estimated with machine vision, the former with an 8 mm lens featuring a wider field of view, and the latter with a 12 mm lens sensing a narrower field of view. As both maps share the same scale, it is clear that higher indices are obtained for the narrow field of view, as expected from the images (Figure 3), but the trend in vegetation growth along the rows is quite similar, similarity that will be statistically determined in Section 6.3. Elevation (Figure 6) and vegetation (Figures 7 and 8) maps, unlike the rest, were automatically generated from the vehicle with no manual intervention, resulting in more data, less subjectivity, and much faster acquisition of key information. For these reasons, automatic perception will always be preferred to manual sampling, and the ideal situation will be when all the traits can be measured robotically from an intelligent sensing unit. So far, many traits require human intercession but the concept of crop biometric maps accepts both data sourcing, permitting its current use in manual mode and its future modernization toward all-automatic perception.

**Figure 9.**
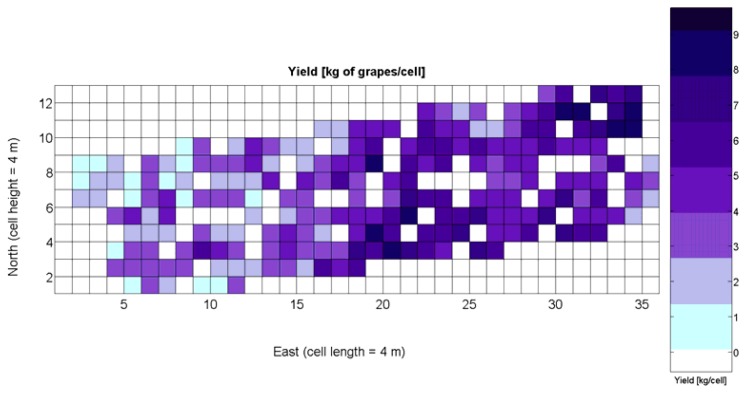
CB map of grape yield (kg of grapes/16 m^2^).

**Figure 10.**
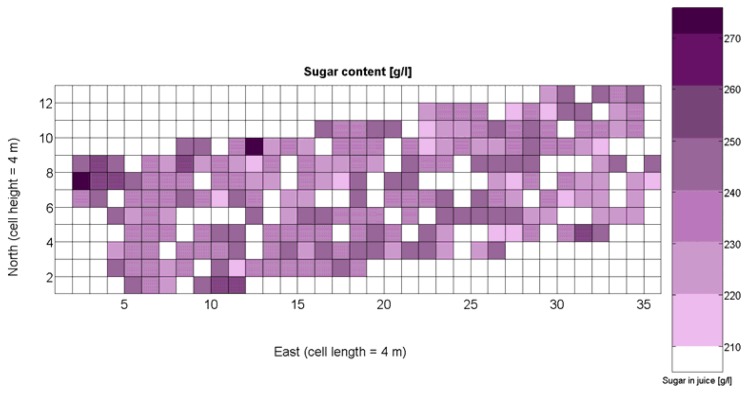
CB map of sugar content in must (° Baumé).

**Figure 11.**
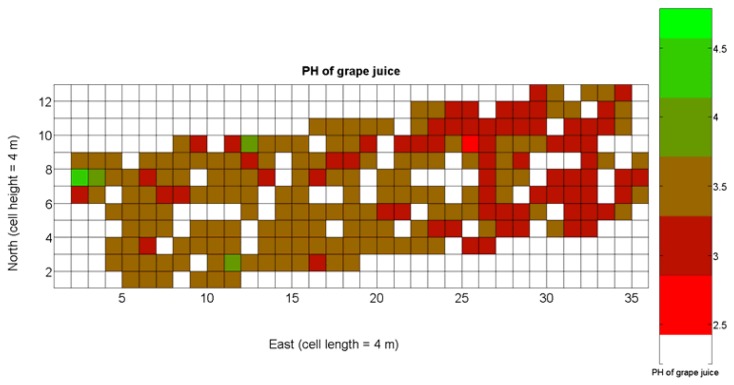
CB map of must pH.

**Figure 12.**
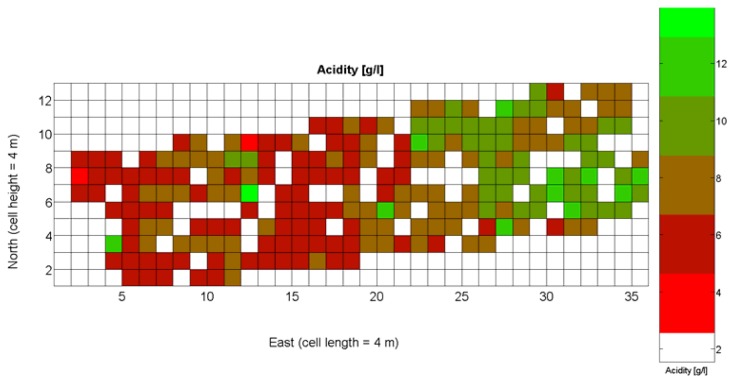
CB map of must acidity (g/L).

**Figure 13.**
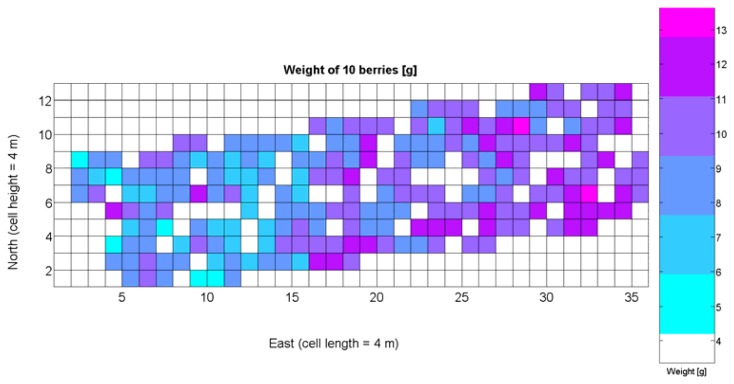
CB map of 10-berry weight (g).

**Figure 14.**
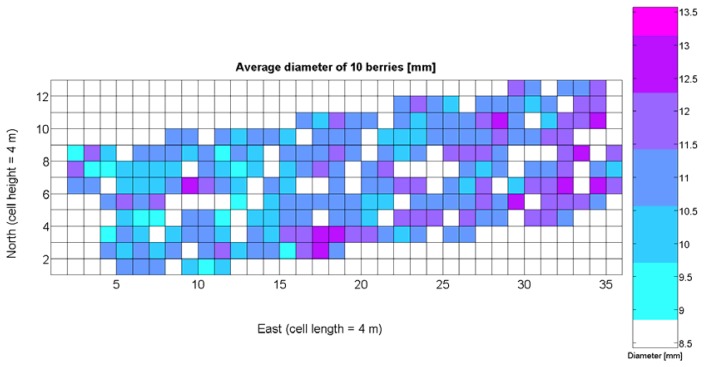
CB map of berry average diameter (mm).

**Figure 15.**
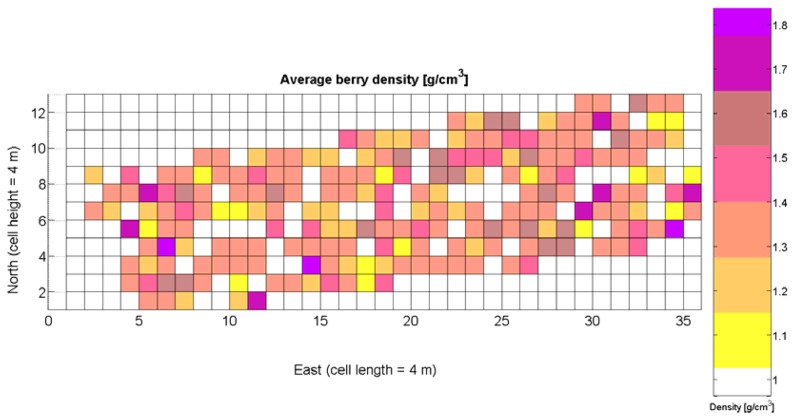
CB map of average berry density (g/cm^3^).

The compendium of maps enclosed in Figures 4, 5, 6, 7 and 8 constitutes the quantization of traits at soil and plant level, as schematized in Figure 1. The rest of the maps belong to the produce level, as they directly relate to the grapes and their juice, technically known as the must. Figure 9 represents the actual yield measured at harvesting time for discrete sections of trellised vine corresponding to the approximate area of a cell (16 m^2^), which in practical terms resulted in 219 measurements distributed along the ten rows. Even though grape production was manually weighted, there exist commercial yield monitors that may be integrated in grape harvesters for the instantaneous estimation of yield, and as a result this trait will likely be measured automatically in the near future. Yield is a principal trait because it is usually both predicted and measured, given its importance for growers and winery managers. Figure 10 provides the sugar content of the must measured in degrees Baumé. The acidity of the must was quantitatively determined through the pH (Figure 11) and with a semi-automatic tritator in g/L according to the distribution of Figure 12. Several physical parameters of the berries were also estimated from the samples taken during harvesting time. Figure 13 maps the distribution of 10-berry weights along the rows, and Figure 14 depicts the average diameter of the sample assigned to each cell. As Cabernet-Sauvignon grapes are quite spherical in shape, their diameter provides a good estimate of their volume, whose relation with weight leads to the indirect estimation of density (Figure 15), easily calculated cell by cell.

### 6.2. Statistical Nature of Selected CB-Traits

**Figure 16.**
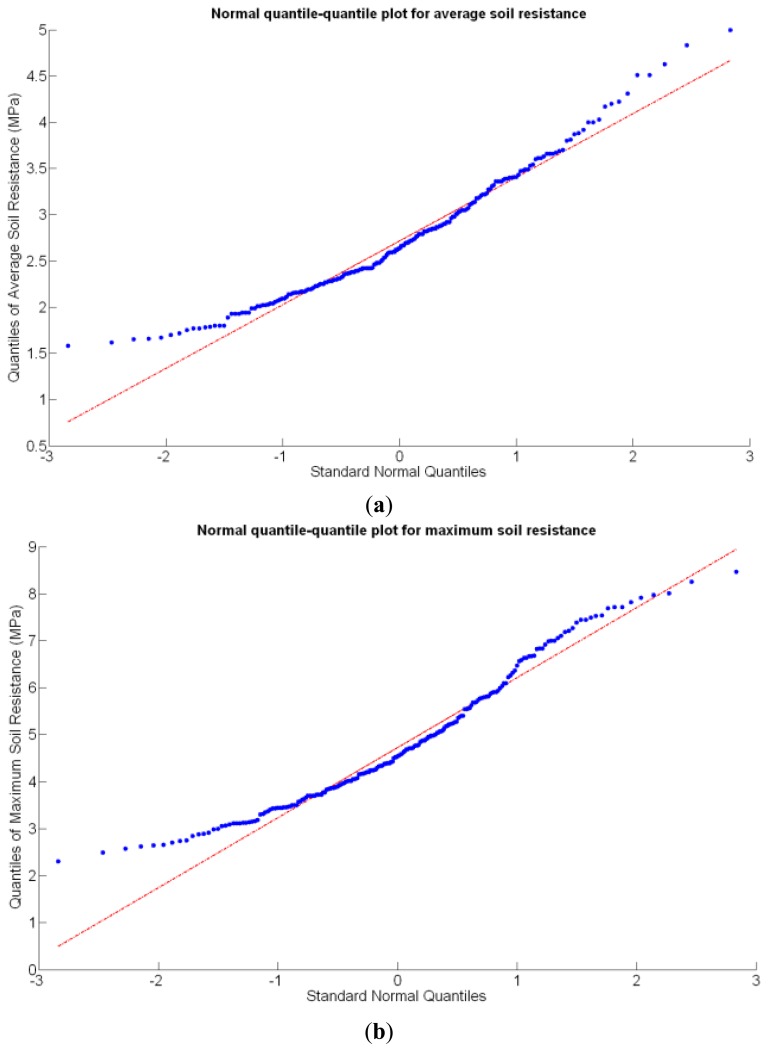
Normal quantile-quantile plots for soil resistance (MPa): (**a**) Average values; (**b**) Maximum values.

**Figure 17.**
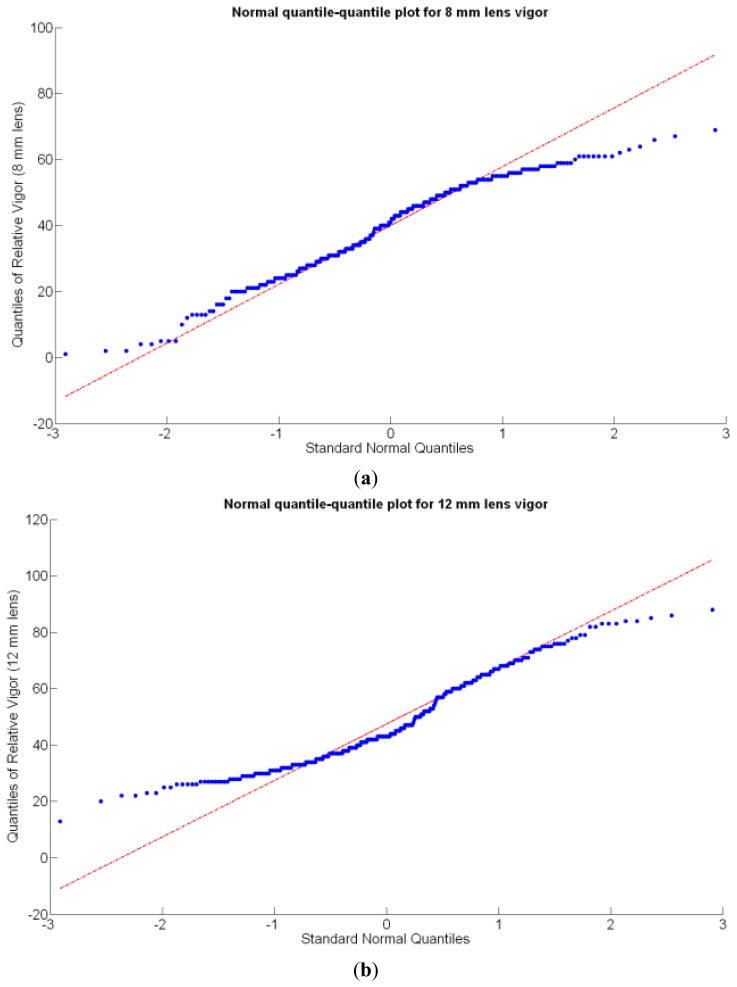
Normal quantile-quantile plots for relative vigor of vines (%): (**a**) V-8; (**b**) V-12.

**Figure 18.**
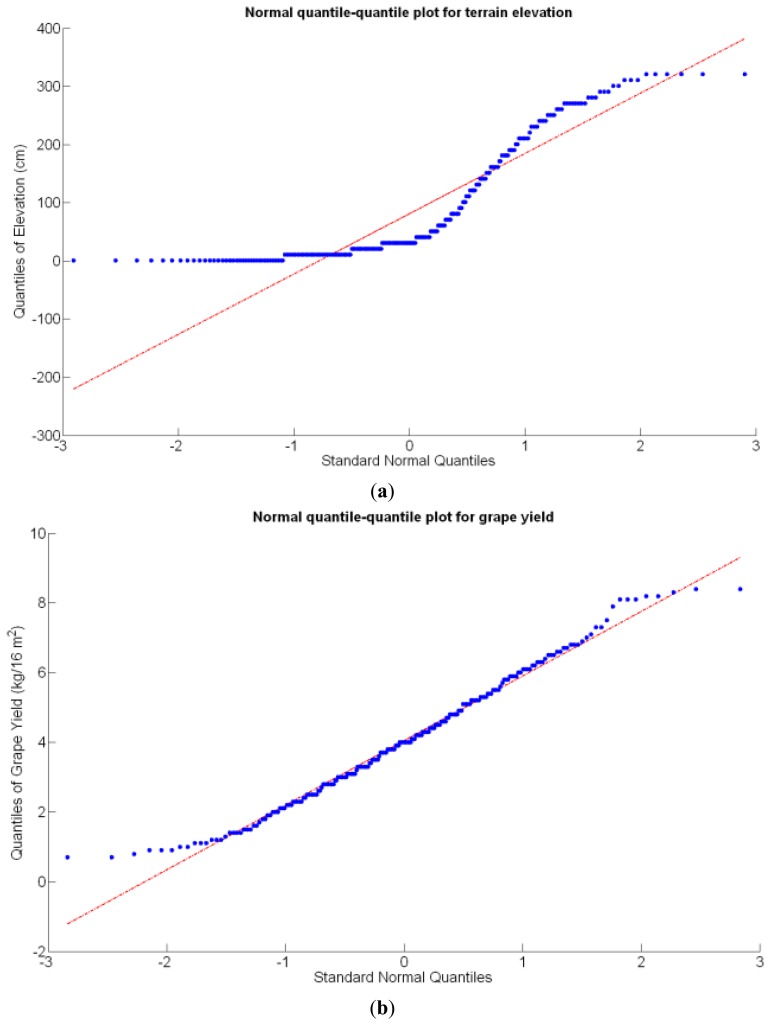
Normal quantile-quantile plots: (**a**) Terrain relative elevation (cm); (**b**) Grape yield (kg/16 m^2^).

**Figure 19.**
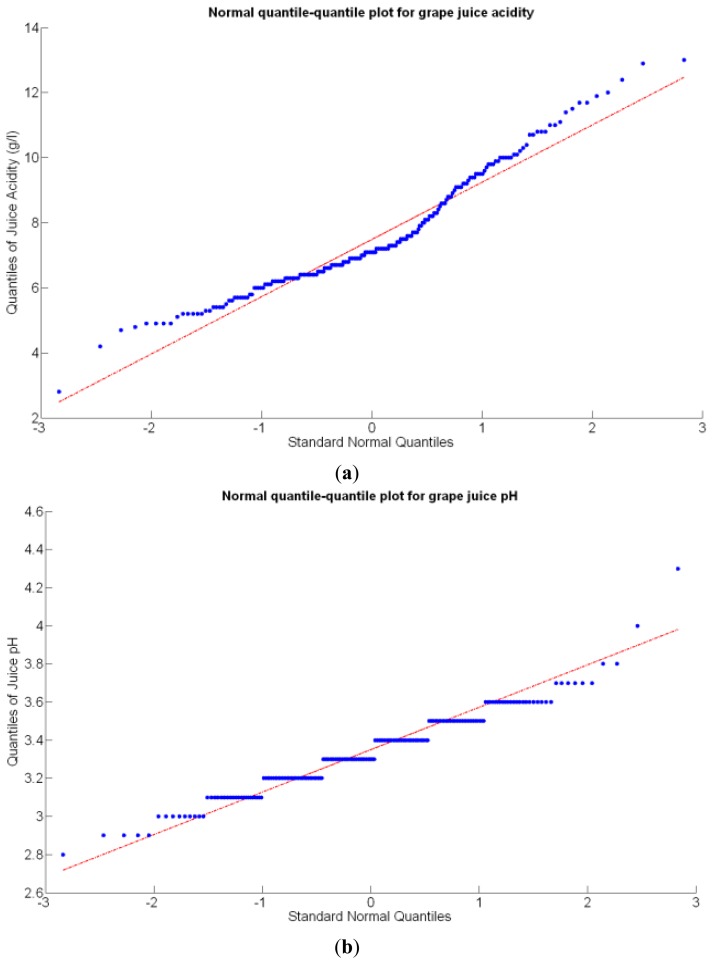
Normal quantile-quantile plots for must acidity: (**a**) Total acidity (g/L); (**b**) pH.

**Figure 20.**
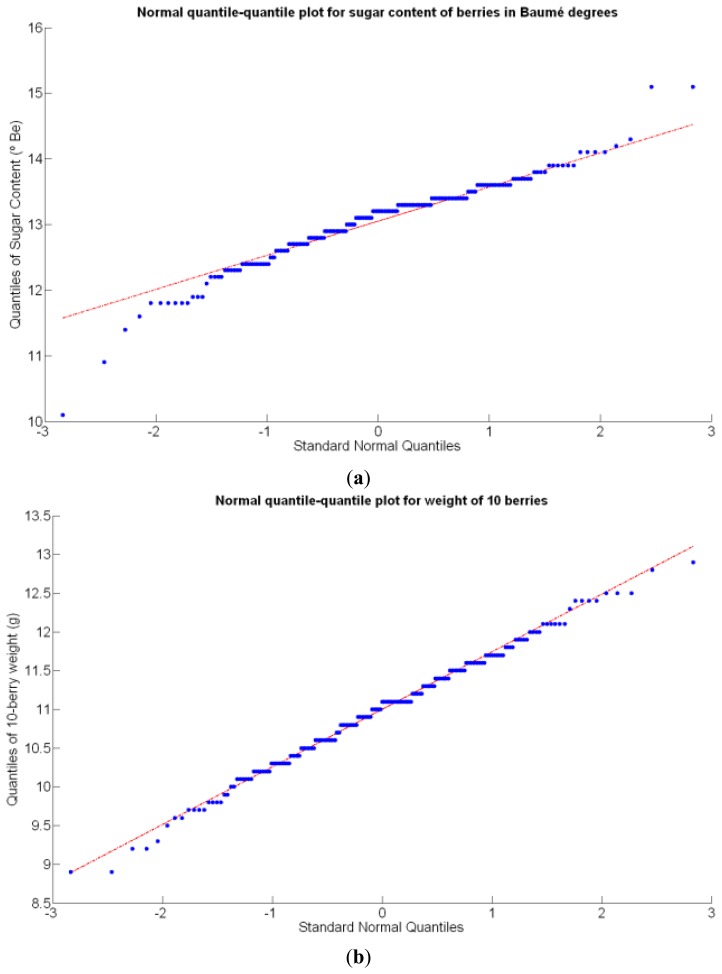
Normal quantile-quantile plots: (**a**) Sugar content in must (° Baumé); (**b**) 10-berry weight (g).

**Figure 21.**
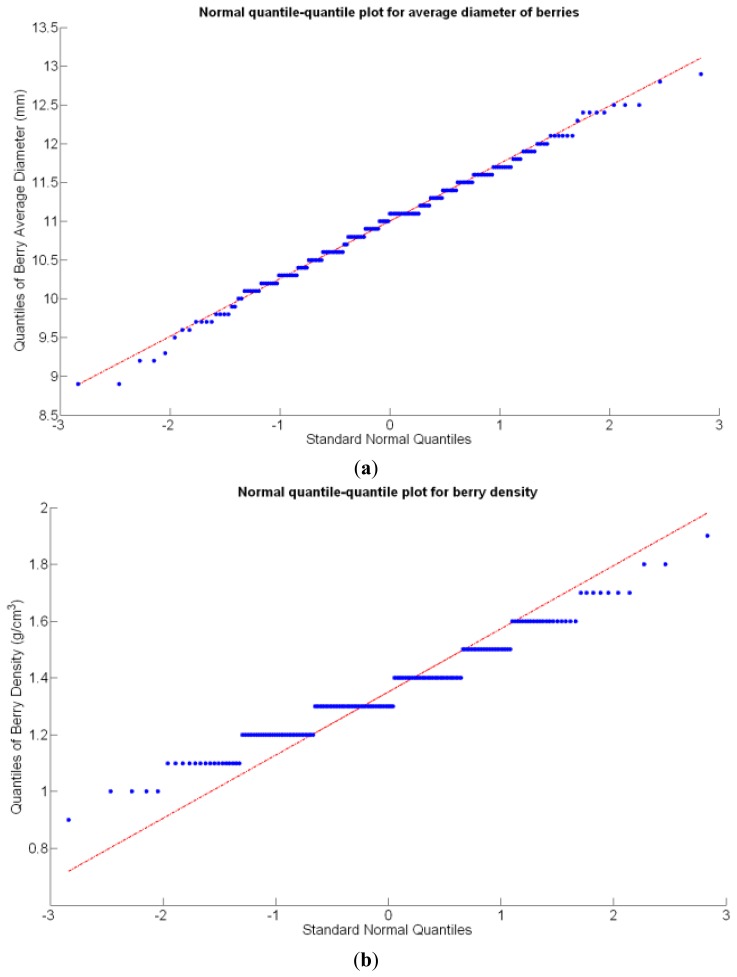
Normal quantile-quantile plots: (**a**) Berry average diameter (mm); (**b**) Berry density (g/cm^3^).

Before conducting inferences on means and standard deviations for the 12 biometric traits considered in the vineyard study, and mapped in Figures 4, 5, 6, 7, 8, 9, 10, 11, 12, 13, 14 and 15, the assumption of normality must be verified. To do so, the normal quantile-quantile plots offer an excellent tool to quantify the statistical validity of the conclusions later drawn from correlation and regression analyses. Figure 16 depicts the quantile plots for the soil resistance to root growth, either average (a) or maximum (b). Similarly, Figure 17 evaluates the normal behavior for the relative vigor, either estimated with an 8 mm lens (a) or through the 12 mm lens (b). Figure 18a depicts the quantile plot for field elevation, and the behavior of yield is examined in Figure 18b. The acidity of the must is checked in Figure 19, directly in g/L (a) and also through the pH (b). The pattern followed by the distribution of sugar content within the field is shown in Figure 20a. The statistical nature of berry physical parameters was studied by analyzing the normality of the distribution of weight (Figure 20b), average diameter (Figure 21a), and berry density (Figure 21b). Table 2 summarizes the main statistical variables for the 12 traits initially considered in the Cabernet-Sauvignon vineyard of Figure 2. The table shows that there are no significant outliers in the data, and the stochasticity of traits is excellent except for the terrain elevation (Figure 18a), which obviously cannot follow a random pattern as it actually represents the profile of the terrain where the vines are planted. This fact must be taken into account if the elevation trait contributes to predictive models.

### 6.3. Coherence between Equivalent or Related Biometric Traits

In the automatic assessment of vine vigor, and from a physical standpoint, the perception of vines with a 12 mm lens (V-12) necessarily fills the image better than its equivalent image taken with the 8 mm lens (V-8), as the former provides a closer look that avoids peripheral distractions such as soil, trellis frames, or vehicle parts. However, the goal of vigor maps is the acknowledgement of the spatial variation of vegetation, and a priori, both estimations (8 mm and 12 mm lenses) should lead to similar conclusions with independence of the measuring scale used. A close look at their variances (Table 2) yields 219%^2^ for the 8-mm assessment and 276%^2^ for the 12-mm estimation, which are diverse enough not to consider the regression fallacy issue explained in Section 5.3.

**Figure 22.**
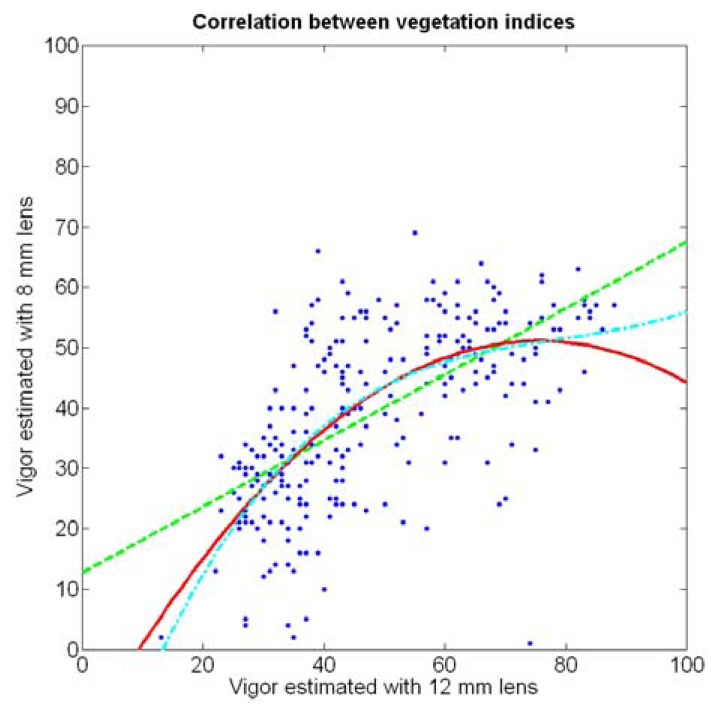
Correlation between alternative vegetation indices V-8 and V-12.

If both variables provide equivalent information on vegetation variability, there must be a conversion equation that translates any given level of foliar coverage to either V-8 or V-12; in other words, there should be a significant correlation between both variables when a regression fit between them is obtained. Due to the high variability found in the field with biometric traits, the resistant coefficient of determination given in Equation 2 results helpful to select the best fit between V-8 and V-12. Figure 22 depicts the scatter plot of V-12 *vs.* V-8 superposed with the linear, quadratic, and cubic fits specified in Table 3.

In addition to featuring the lowest *R^2^* and $R_{\text{res}}^{2}$ values (Table 3), the linear fit produces values of V-8 higher than V-12 for small vegetation coverage, which is incorrect from a physical point of view. The quadratic and cubic regression both offer a more realistic model, and although the small improvement obtained with the cubic version in terms of coefficients of variation may not justify such degree of complexity, the behavior of the cubic model for values of V-12 greater than 70% is clearly superior, as this model always keeps V-12 > V-8, which is what happens in reality due to the morphology of the images.

**Figure 23.**
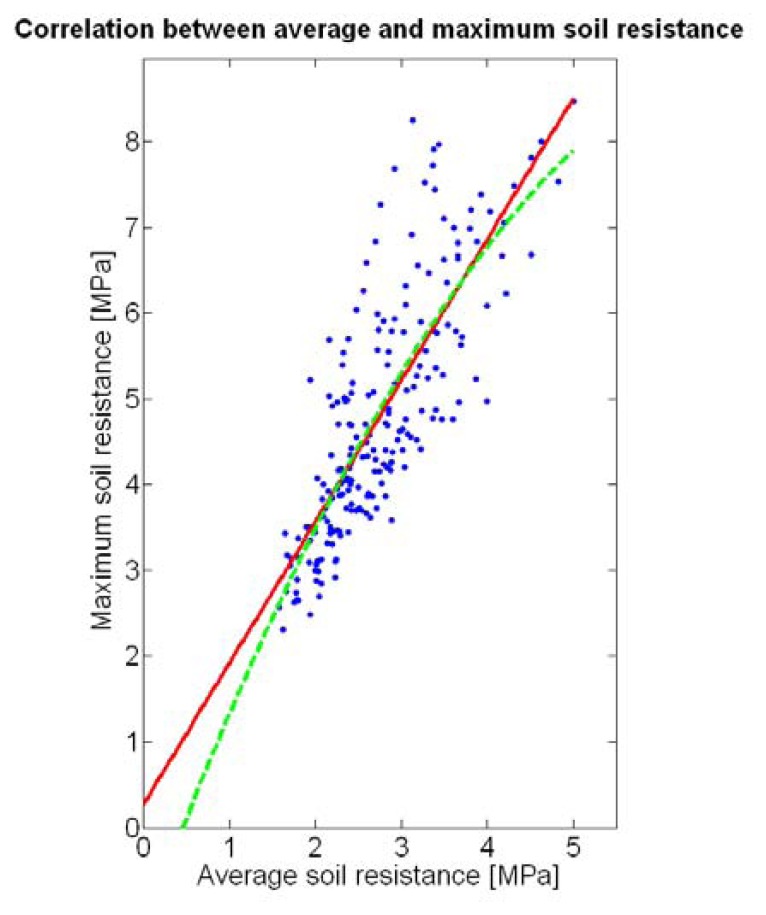
Correlation between average and maximum soil resistance.

The compactness of soil may complicate the proper development of vine roots, and in consequence limit the optimum growth of plants and fruits. As happened with the assessment of vegetation, soil resistance was also estimated through two alternative measurements: average resistance and maximum resistance. From the physical standpoint, which must be always in sight, there must be a significant correlation between both traits. In fact, it is reasonable to think that only one of them will suffice to build prediction models if soil resistance turns out to be determinant in the prediction of yield or quality, as both indicators supply equivalent information. *A priori*, no preference can be established between average or top values until the enunciation and detailed analysis of predictive models. The comparison of variances (Table 2), however, yields a superiority of average values (σ^2^ = 0.46) in comparison with maximum values (σ^2^ = 1.96). As variances are notably different, the regression fallacy is not a problem in the construction of regression models. Figure 23 plots the average soil resistance *versus* the maximum soil resistance.

This time there is no benefit in using a quadratic fit, as linear regression is equally good and clearly simpler. The dispersion of data is milder than in Figure 22, and consequently the differences between *R^2^* and $R_{\text{res}}^{2}$ (Table 4) are much smaller for both fits. Should outliers have appeared in the data, the latter would have provided a better assessment of the fitting quality.

### 6.4. Prediction Models for Grape Yield

Yield is one of the capital traits measured and studied in the biometry of vineyards, as it facilitates the construction of predictive models on future production from other traits involved in the process and considered predictor traits. From a physical perspective, field experience in agronomy has shown that high soil resistance to root penetration is not favorable to the development of roots, which in turns can limit the production of leaves, flowers, and fruits, although vines have traditionally endured poor terrains; the accumulation of water in the soil is positive for the development of the canopy, inducing higher yields; and the direct assessment of foliage in grapevines has been traditionally related to grape yield. In most cases, the soil and plant-level biometric traits, which can be measured way ahead harvesting time, possess the capacity to contribute in the prediction of upcoming yields. However, the following intricacies appear with the biometric traits considered in this study:

1. Two different optical architectures have been implemented to quantize plant vigor, namely V-8 and V-12. As expected, both estimations are related, but nonlinearly according to Figure 22 and Table 3. Fortunately, both behave as normal variables although their variances are significantly different (Table 2). Statistical evidences will be necessary before determining which variable—or even both–should be included in the final predictive model.
2. Likewise, the soil resistance to root development has been estimated from average and maximum measurements of standard penetration tests, which are linearly related according to Table 4. Figure 16 shows that the assumption of normality is met for both measurements, but further statistics are needed for their potential consideration as predictors.
3. The water content in the soil was not directly measured; instead, it was indirectly assessed by the relative elevation of the plants with respect to the lowest zone of the field, where water accumulates as a consequence of runoff. As a result, the highest areas of the field are generally drier than the lowest headlands of the field, as indicated in Figure 6. This variable, in addition, behaves non-linearly and does not follow a normal distribution according to Figure 18a, thus special caution must be taken when using it with conventional statistics such as least squares estimators of regression models.

Once the statistical nature of potential traits has been determined and special situations such as violations of the normality assumption or the presence of alternative measurements have been considered, predictive models may be enounced, compared, and scored. The selection of the best model is subjected to the following criteria: the largest coefficients of variation *R^2^* and $R_{\text{res}}^{2}$ as defined in Equations (1) and (2); the largest *F-statistics* from the ANOVA table of multiple regression analysis; the lowest *p-value* from ANOVA; and the analysis of *t-statistics* for collinearity and individual trait significance. In the comparison of models, the coefficients of variation should be adjusted because not all the models include the same number of predictor variables. However, Table 2 shows that the number of measurements above 200 is large enough to palliate negative effects, and in addition, the implementation of a resistant coefficient $R_{\text{res}}^{2}$ adds robustness to the model selection. All the calculations carried out in this article have been performed with a 95% confidence interval, which is quite high for the actual variability found in the field. The composition of predictive models for grape yield proceeds from the combination of the following principles: the empirical judgment and theoretical considerations on the vineyard crop; the stepwise selection of predictor traits, either adding or backward suppressing; and the assessment of collinearity effects. Table 5 provides the key details of the 19 models proposed for yield prediction, where *X_1_* represents the vigor V-8 (%), *X_2_* the vigor V-12 (%), *X_3_* the elevation at which the plant is located (cm), *X_4_* the average soil resistance (MPa), *X_5_* the maximum soil resistance (MPa), *y* the yield predicted by the model (Kg/16 m^2^), and *Z_3_* the standardization of *X_3_* with Equation (3) and the data of Table 2:

(3) $$Z_{3}=\frac{X_{3}-{\bar{X}}_{3}}{\sigma }=\frac{X_{3}-85.3}{97.3}$$

As all traits at soil and plant level (Figures 4, 5, 6, 7 and 8) may potentially affect the upcoming yield, the first attempt constructing the yield model considers all predictors from *X_1_* to *X_5_*. Model 1Y in Table 5 represents this option. The results of the ANOVA for Model 1Y are printed in Table 6. In addition to the second lowest F-stat, the t-statistics for predictor variables indicate that *X_2_*, *X_4_*, and *X_5_* do not add significant value to the model, and therefore may be excluded.

The results of Table 6 imply that soil measurements are not helpful in the prediction of yield. However, the effect of the soil might be masked by the interference of the rest of predictor traits considered in Model 1Y. In order to clarify it, Model 5Y (Table 5) was proposed with the purpose of analyzing the effect of the average soil resistance (*X_4_*) and its interference with the relative vegetation assessed with *X_1_*, which is significant in Models 1Y to 4Y. Table 7 shows the results of the ANOVA for Model 5Y, where the lack of significance for the soil resistance and its possible interference with vegetation is evidenced again. The F-statistics of Model 5Y shows poor results as well, confirming the exclusion of soil resistance traits from the definite model.

**Figure 24.**
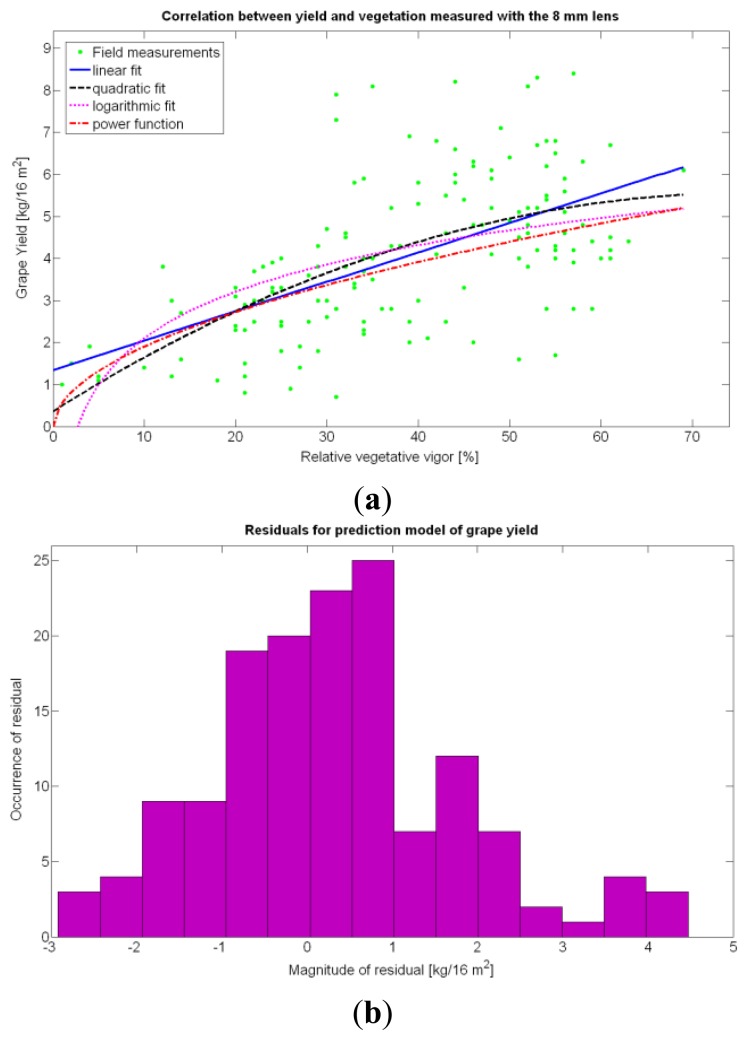
(**a**) Prediction models for yield as a function of V-8 relative vigor; (**b**) Residuals for predictive Model 15Y (power function).

Even though the elevation (*X_3_*) seems to be well correlated with yield, as indicated by the F-stat and $R_{\text{res}}^{2}$ of Models 9Y and 13Y, the strong rejection of the term *X_1_ ·X_3_* given by the t-stat of Model 6Y suggests that both variables might be *collinear*. In order to investigate a potential source of collinearity between these predictors, and knowing that *X_3_* does not behave as a normal distribution, *X_3_* was standardized through Equation (3) and incorporated as *Z_3_* into Model 18Y of Table 5. Nevertheless, the standardization of *X*_3_ did not result in any benefit for the model, and the only trait, out of the set measured in this application, that seems to correlate well with grape yield was *X_1_*. The choice finally selected was Model 15Y, with the ANOVA results of Table 8. The main reasons for this preference are:

1. Highest $R_{\text{res}}^{2}$.
2. Highest F-stat.
3. All terms in the model are 95% significant according to the t-stat of Table 8.
4. Simplicity and easiness of use, as shown in the model definition of Table 5.
5. The model is valid in the entire domain of variable *X_1_* (V-8 vigor), which ranges from 0% (no vegetation) to 100% (full coverage). It makes (physical) sense for no vegetation, as y(0) = 0, meaning that if there is no vegetation, there is no yield. However, the model underestimates yield for high vigor because y(100) = 6.3 kg/16 m^2^, and there are cells up to 8.4 kg/16 m^2^ (Figure 9), accounting for an error of 25%.
6. The residuals, plotted in Figure 24b, behave quite *normally*.

The quadratic and cubic models involving *X_1_*, as well as the logarithmic fit, do not bring advantages that justify their selection. The linear fit, in addition, does not provide reasonable results for *X_1_* = 0. The alternative fits for yield prediction involving *X_1_*, namely Models 8Y, 11Y, and 14Y, are represented in Figure 24a.

Overall, the best fit given by the power function of Model 15Y yields an $R_{\text{res}}^{2}$ of 0.5, which in general terms cannot be considered strong, as it only explains 50% of variability. Yet, these results are expected from biological systems due to their high variability, as shown in the scatter plot of Figure 24a. It is expected that better measurements of traits and more replications conducted in the same field year after year will eventually lead to model fits significantly better.

### 6.5. Prediction Models for Grape Quality and Enological Potential: Quality Potential Index (QPI)

Unlike yield, which is a trait easy to measure, there is no such trait called grape quality. Yet quality is a key factor, if not the most determinant nowadays for winemakers, because a reduction on yield can be counterweighted by an excellent quality, but it never works the other way around, as a drop in quality affects the reputation of the brand and may have negative consequences for many years regardless of yield. The key question is how to define the term quality with regards to grapes, and consequently to prospective wine. As a matter of fact, it seems more reasonable to speak of *potential quality*, as the developed models try to predict the quality of the future wine based upon data recorded in the field from plants and grapes in growing stages ranging from *véraison* to harvesting time. Roger Pellenc, the French manufacturer of grape harvesters, states [22] that the base characteristics of the vine are essentially the quantity of grapes harvested, the sugar of the grapes, their acidity, and the health status of the plant, which is understood as the growth of vine shoots during the vegetative period.

Before defining an index for quality potential as a function of the traits and measurements available from the CB maps developed, it is necessary to find out what viticulturists and winemakers consider it to be the ideal balance for grapes at the time of harvesting, that is, determining the perfect ripeness for obtaining the best possible wine. The frequent update and tracking of certain maps may lead to associate certain tastes with certain changes in measured factors. According to Cox [23], the factors to be measured are degrees Brix, titratable acidity in g/100 mL (TA), and pH, with target readings for perfect red grapes of 22 Brix, 0.75 acid, and a pH about 3.4. Brix degrees provide the percentage of sugar in the grape juice, whereas acids give crispness, brightness, and thirst-quenching qualities to wines, being essential components of the balance in a fine wine. On the other hand, pH is related to TA but differs from it and may or may not be correlated with the amount of tartaric acid of grape juice. The ideal value of pH is 3.4 for red wine, but it may be higher even when TA is within the optimum range.

The ratio of Brix to TA is a better indicator of ripeness and quality than sweetness or tartness alone. Researchers at the University of California at Davis [23] have found that wines are properly balanced when Brix:TA is between 30 and 35, and preferably 30. An even more accurate measure for quality sets the optimal situation when Brix times pH^2^ approaches 260 for red wines or 200 for white wines. The list of traits measured from the grapes and represented in the crop maps of Figures 10, 11 and 12 allow for the implementation of these quality indicators in the quest of a general quality index that can take part in predictive models within the scope of crop biometrics. In particular, let *X_6_* be the sugar content measured in degrees Baumé (Figure 10), *X_7_* the total acidity in g/L (Figure 11), and *X_8_* the must pH (Figure 12), the sugar content in degrees Brix (*X_9_*) and the titratable acidity in g/100 mL (*X_10_*) can be easily calculated with Equations 4 and 5:

(4) $$X_{9}=1.8\cdot X_{6}$$

(5) $$X_{10}=0.1\cdot X_{7}$$

Taking the nomenclature used for the CB traits measured in the vineyard to the recommendations given above by Cox and the University of California [23], the expressions given in Equations (6) and (7) must hold for the optimum quality of Cabernet-Sauvignon grapes:

(6) $$\frac{X_{9}}{30\cdot X_{10}}\approx 1$$

(7) $$\frac{X_{9}\cdot X_{8}^{2}}{260}\approx 1$$

The multiplication of Equations (6) and (7) leads to the expression of Equation (8), that permits the evaluation of quality from the predictor traits estimated in the field and mapped in Figures 10, 11 and 12.

(8) $$\frac{X_{9}}{30\cdot X_{10}}\cdot \frac{X_{9}\cdot X_{8}^{2}}{260}=\frac{{\left(X_{8}\cdot X_{9}\right)}^{2}}{7800\cdot X_{10}}\approx 1$$

The expression deduced in Equation (8) is the basis for measuring the quality potential in a new CB map. However, pivoting around 1 as the ideal quality is not convenient because values will grow unevenly according to whether they are above or below 1. In order to circumvent this issue and establish a more symmetrical distribution around zero (maximum quality), the *Quality Potential Index* (QPI) was defined by taking common logarithms to Equation (8), as shown in Equation (9):

(9) $$\text{QPI}\overset{\text{def}}{=}\log_{10}\frac{{\left(X_{8}\cdot X_{9}\right)}^{2}}{7800\cdot X_{10}}=2\cdot \log_{10}(X_{8}\cdot X_{9})-\log_{10}(7800\cdot X_{10})$$

The application of the natural logarithm to Equation (8) would have expanded the scale, but with the use of common logarithms in base 10, the working scale for quality potential is practically limited to the interval [−1,1], which facilitates the use and interpretation of QPI maps, the CB map version of grape quality. Given that total acidity was mapped in g/L in Figure 11, and represented by *X_7_*, the final expression for QPI should use *X_7_* rather than *X_10_*, which can be easily performed by introducing Equation (5) into Equation (9). The final expression for the QPI is given in Equation (10), where *X_8_* is the must pH, *X_7_* is total acidity in g/L, and *X_9_* is the sugar content in degrees Brix. The best quality will be obtained for QPI values around zero (log_10_ 1 = 0), moving further away as quality decreases; so, for the optimal situation recommended by Cox of *X_7_* = 7.5 g/L, *X_8_* = 3.4, and *X_9_* = 22, the QPI is 0.019, which in practical terms can be considered as 0:

(10) $$\text{QPI}\overset{\text{def}}{=}2\cdot \log_{10}(X_{8}\cdot X_{9})-\log_{10}(780\cdot X_{7})$$

**Figure 25.**
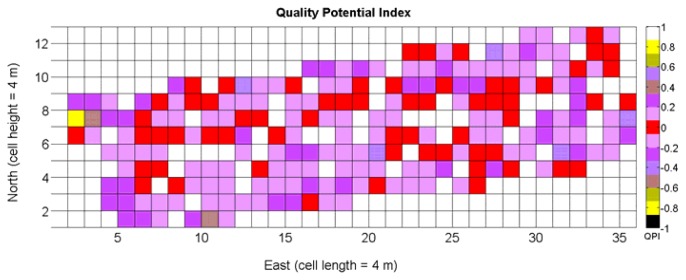
CB map for the Quality Potential Index QPI.

The QPI map resultant from applying Equations (4) and (9) to the CB maps of Figures 10, 11 and 12 is plotted below in Figure 25. Inference statistics for a total number of 219 cells lead to a maximum QPI of 0.796 and a minimum of −0.416, both in the range [−1,1] as normally expected. The average is 0.031 and the median 0.051, with a standard deviation of 0.1698.

**Figure 26.**
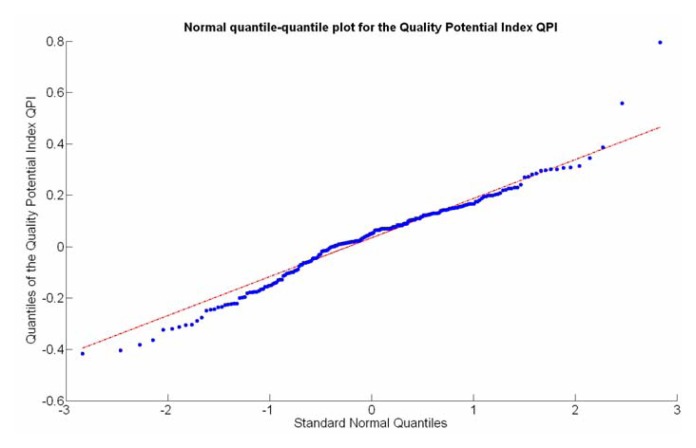
Normal quantile-quantile plot for the Quality Potential Index QPI.

The QPI values assigned to the cells forming the vineyard rows highlight the optimum quality for magnitudes around zero, represented in the map by red cells. As values move further away from zero, either positive or negative, the quality of grapes determined at harvesting time decreases. The maximum value of QPI in the map is about 0.8, so, for convenience, a value of 1 was assigned to cells without information in order to ease the interpretation of the QPI map. All the variables (predictor traits *X_7_*, *X_8_*, and *X_9_*) used in the definition of QPI (Equation (9)) are normally distributed according to Figures 19 and 20a, but due to the fact that this definition involves the product of variables and the use of logarithms, the normality assumption for QPI must be carefully checked. Fortunately, the quantile-quantile plot of Figure 26 proves that QPI behaves as a normal distribution with an excellent match to standard normal quantiles. Once the QPI has been defined as the systematic procedure to quantify quality potential for the future wine, expressed as a combination of objective measurements available from CB maps, the next stage consists of predicting QPI from crop traits available before or at harvesting time. As occurred for the prediction of yield, multiple models need to be evaluated, each one featuring diverse traits, in order to select the most appropriate model to estimate spatial variations of grape quality in the field. The previous experience with yield predictive models and some preliminary tentative trials suggest not to consider vigor V-12 (*X_2_*), soil properties (*X_4_* and *X_5_*), and elevation (*X_3_*) due to their poor contribution to the prediction of grape production.

Table 9 summarizes the statistical evaluation of the 13 models proposed to predict quality in QPI format, where *X_1_* is vigor V-8 (%), *X_y_* is the actual yield (kg/16 m^2^), *X_11_* is the weight of 10 random berries (g), *X_12_* is the average diameter of the berries (mm), and *X_13_* is the average berry density (g/cm^3^).

**Figure 27.**
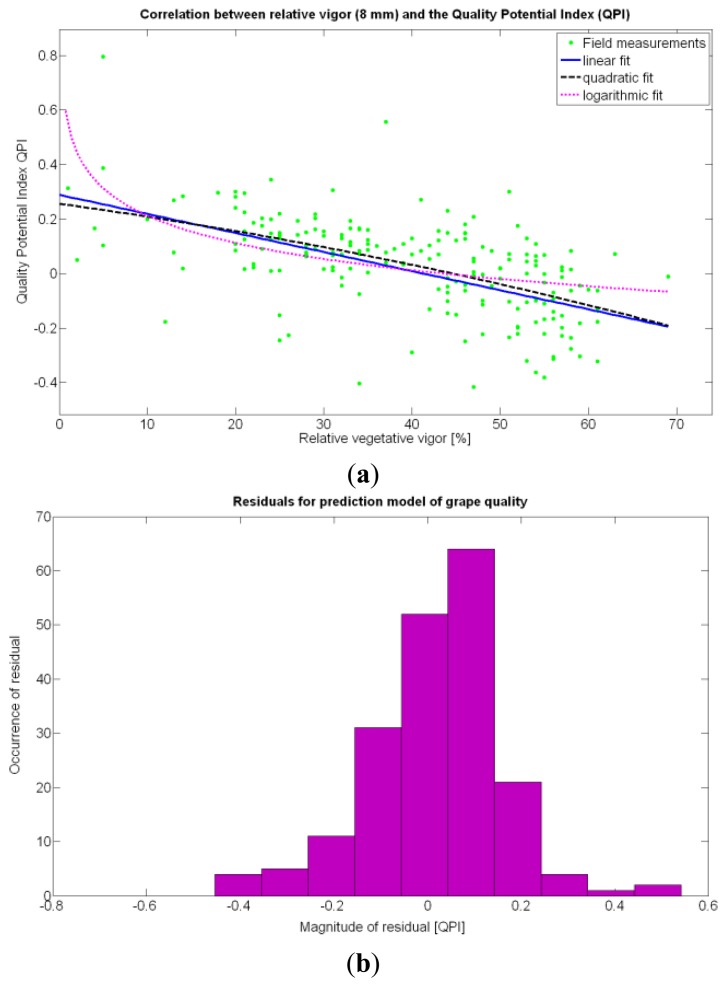
(**a**) Prediction models for QPI as a function of V-8 relative vigor; (**b**) Residuals for predictive Model 3Q (linear fit).

According to Table 9, the goodness of the 13 fits proposed for quality predictions is, generally speaking, weaker than for yield predictions; yet interesting conclusions can be withdrawn from their analysis. The statistical examination of the models clearly indicates that the quality of grapes, understood as the optimal balance between acidity and sugar content at harvesting time, is independent of the size, weight, and density of the berries. Furthermore, based on these results, it does not depend on the yield but can be correlated to vine vigor in such a way that optimal quality occurs with a moderate vegetative development of the vines, as shown in Figure 27a. In fact, excessive foliar growth has a negative influence, numerically set by the QPI, on grape quality, something that was already known in broad terms by viticulturists and growers, but that can be mathematically determined through this method. The main conclusion of the QPI prediction model is that berry quality mostly depends on the relative vigor of the plants, and even though the variability in the field is very high, an improved assessment of vine foliar growth will surely lead to more precise predictions of quality before harvesting. The multivariate analysis summarized in Table 9 consistently rejects all the variables (predictor traits) different from *X_1_* as influencing the quality potential index QPI. In coincidence with the yield predictor model selected (15Y), the spatial variability of vegetation growth is, by far, the most influential parameter to track in the management of vineyards, especially when it is performed on a strong technological basis.

The statistical indicators of Table 9 point to the conclusion that the best way of predicting quality is from the relative vigor V-8 estimated with *X_1_*, although this relationship can be linear or nonlinear. Figure 27a shows that there is no much difference between the linear, quadratic, and logarithmic fits. However, in addition to the highest F-stat and coefficient of determination, the linear model is always easier to use for its simplicity, and therefore Model 3Q will be the selected choice. The residuals of the data after applying Model 3Q behave quite close to a normal distribution, as plotted in Figure 27b. This model yields a QPI of 0.289 for no vegetation (*X_1_* = 0%), which indicates low quality; but also produces a QPI of -0.41 for full coverage (*X_1_* = 100%), which represents very poor quality. Model 3Q allows, too, the calculation of the relative vegetation *X_1_* that leads to the maximum quality QPI = 0. This value corresponds to 41%, foliar coverage that can be taken to the V-8 map of Figure 7 to discover that these cells correspond to the west side of the field, with higher elevation, less vegetation, less yield, and less water content in the soil.

### 6.6. Impact of Crop Biometric Maps Resolution on Predictive Models

**Figure 28.**
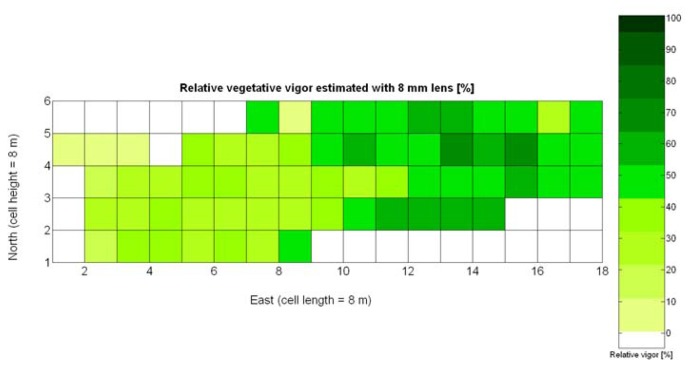
Low resolution CB map of relative vine vigor estimated with an 8 mm lens: V-8 (%).

**Figure 29.**
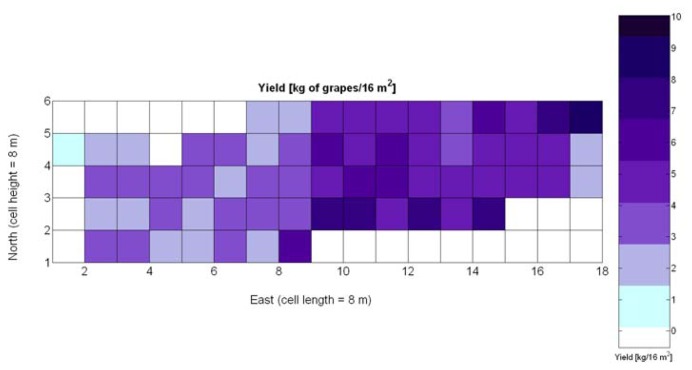
Low resolution CB map of grape yield (kg of grapes/16 m^2^).

One of the advantages that make global grids and CB maps powerful is the capability to adjust the resolution of the maps to the needs of the user. However, the invariance of prediction models for diverse resolutions cannot be taken for granted unless there is a proof of the model's validity for alternative sizes of the cells. As a result, it is important to determine to what extent predictive models change when resolution varies. To do so, the default cells of 4 m × 4 m were doubled in both dimensions to increase the working area fourfold and become square cells of dimension 8 m × 8 m. Figure 28 shows the relative vegetative vigor V-8 (%) with the new resolution, and Figure 29 represents the low resolution version of the yield map. As the working area has augmented four times, several field measurements coincide in each cell to be averaged, and as a result there are no gaps indicating cells without field data as occurred in Figures 7 and 9, which represent the same data at higher resolution. Table 10 specifies the same 19 yield prediction models formulated in Table 5 but adapted to the new resolution, where the meaning of the variables *X_i_* is the same defined for Table 5 but relative to the low resolution maps. Note that in spite of increasing the working area fourfold, yield measurements are kept in kg per 16 m^2^ to ease the comparison between maps of different cell size.

**Figure 30.**
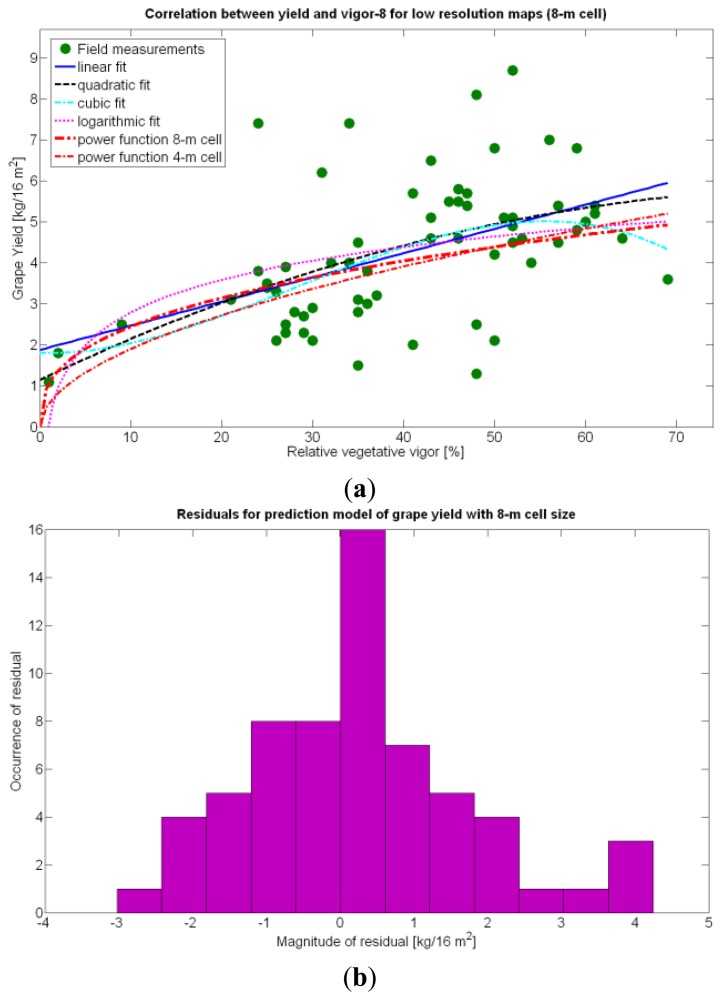
(**a**) Prediction models for low resolution CB maps of yield as a function of V-8 relative vigor; (**b**) Residuals for predictive Model 15Y§ (power function, Table 10).

The immediate apparent fact of downgrading CB map resolution has been a significant increase of the $R_{\text{res}}^{2}$, as evidenced by Table 10. The significant reduction in the amount of data has resulted in the reduction of variability, which in turns has led to a slightly better fit with elevation *X_3_*. Yet, the correlation of yield with relative vigor *X_1_* is equally good, and taking into account the significance of traits (predictor variables) in the real field, the best alternative seems to model yield predictions from vegetation (*X_1_*). Additionally, the assumption of normality cannot be assumed for *X_3_* and there are collinearity issues between *X_1_* and *X_3_* (proved by t-stat in Models Y3§, Y6§, and Y18§). As a result, a fitting equation with *X_1_* as predictor has to be found and compared to Model 15Y. As occurred with high resolution maps and Figure 24a, Figure 30a shows tiny differences between the five models applied; yet, the best performance belongs to the power function of Model 15Y§, as it possesses the highest F-stat and is coherent with nature for *X_1_* = 0. Notice, however, that the magnitudes of the parameters in the equation have changed with respect to Model 15Y. The new model predicts a yield of 5.6 kg/16 m^2^ for full coverage *X_1_* = 100, which is slightly lower than the 6.3 kg found for high resolution maps. The residuals of applying Model 15Y§, plotted in Figure 30b, also follow a normal distribution shape. In addition to alternative fits, Figure 30a provides a graphical comparison between the power functions of Models 15Y and 15Y§. Both functions trace close paths, with larger discrepancies below *X_1_* = 30%.

**Figure 31.**
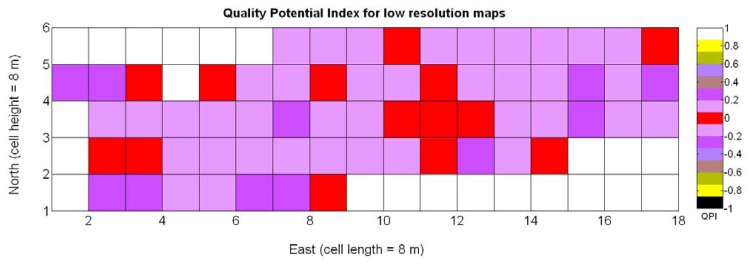
Low resolution CB map for the Quality Potential Index QPI.

The possible variations of the predictive model for QPI (3Q in Table 9) when map resolution is lowered from 35 × 12 cells to 17 × 5 cells were studied following the same procedure outlined for the prediction of yield. Figure 31 shows the low resolution map for the quality potential index QPI, and Table 11 lists the 13 new models for predicting QPI that result from increasing the cell size to 8 m × 8 m. The meaning of the predictor variables featured in the models coincides with that of Table 9.

**Figure 32.**
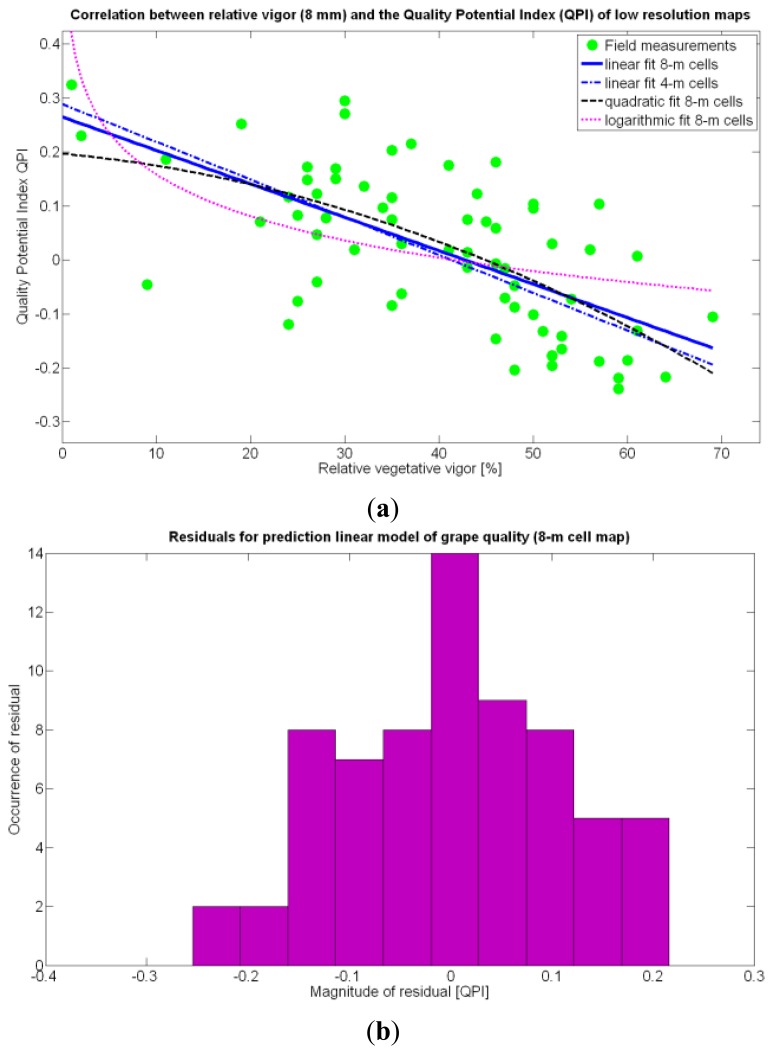
(**a**) Prediction models for low resolution CB maps of QPI as a function of V-8 relative vigor; (**b**) Residuals for predictive Model 3Q§ (linear fit, Table 11).

As happened with yield prediction, results do not change significantly with the modification of the map resolution. Again, there is an improvement of *R^2^* and $R_{\text{res}}^{2}$ induced by the reduction of the dataset, but the linear model 3Q§ still represents the best fit and highest F-stat. In the same fashion, yield and berry density are always rejected from the models by the statistics given in Table 11. Figure 32a confirms that the new linear fit (Model 3Q§) is in reality very close to the high-resolution fit (Model 3Q) and not too far from the quadratic fit (Model 10Q§). Overall, the principal conclusions remain and the optimum quality (QPI ∈ [−0.1,0.1]) occurs with a medium vigor index *X_1_* between 30% and 60%, decreasing in (QPI) quality for vegetation indices above 60%. According to linear Model 3Q§, the best quality (QPI = 0) is found for relative vegetation *X_1_* = 43%, which is very close to the high-resolution value of 41%. The distribution of residuals depicted in Figure 32b reasonably reproduces a normal distribution profile.

### 6.7. Growers Expectancy based on Crop Biometric Models

**Figure 33.**
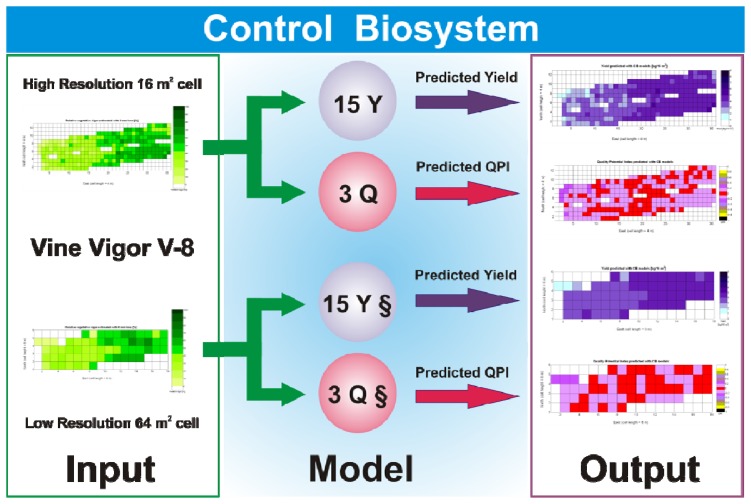
Concept of Control Biosystems for vineyards based on Crop Biometric Maps.

The standard language of crop biometrics consists of maps, thus all predictions formulated within this framework have to be delivered in such format for producers to share, use, and make strategic decisions. As a result, the mathematical body developed hitherto must be integrated in compatible maps. Based on the results found and available field data on vineyard biometrics, and inspired in MIMO control systems, a *Control Biosystem* may be defined such that the input to the system is the relative vegetative vigor estimated with the 8 mm lens (*X_1_*) between *véraison* and harvesting, and the output comprises the prediction maps of yield and quality standardized by QPI. This biosystem can be applied to either high or low resolution maps as schematized in Figure 33.

**Figure 34.**
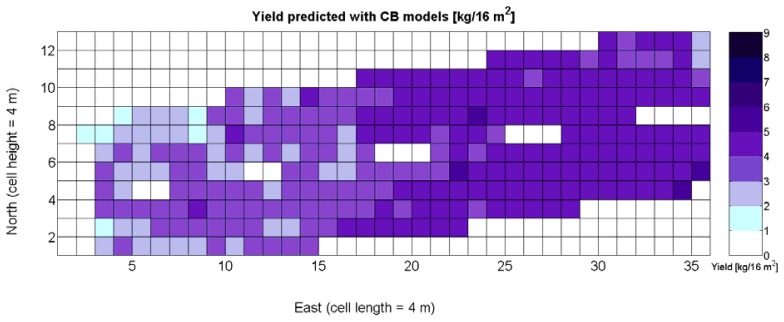
Yield predicted with CB models for high resolution maps.

**Figure 35.**
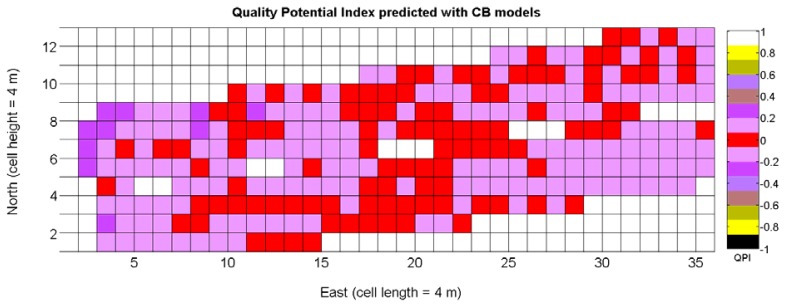
Quality Potential Index QPI predicted with CB models for high resolution maps.

**Figure 36.**
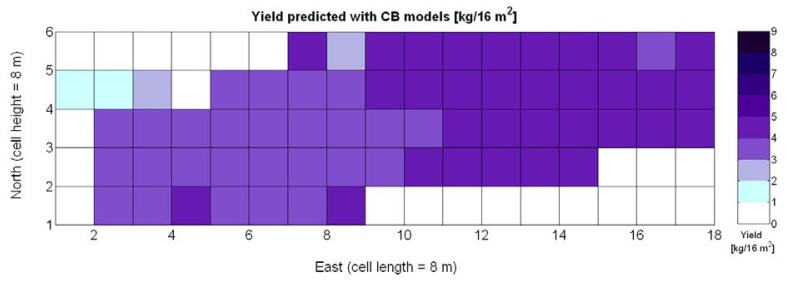
Yield predicted with CB models for low resolution maps.

**Figure 37.**
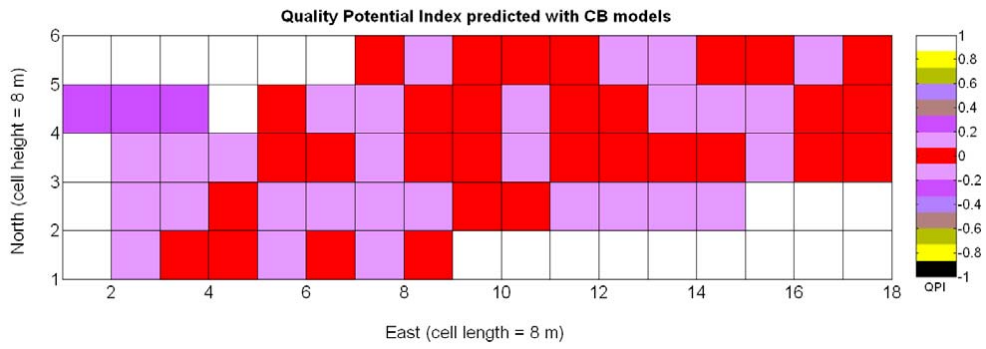
Quality Potential Index QPI predicted with CB models for low resolution maps.

Figure 33 provides a practical example of how to apply the idea of crop biometrics to vineyard production with the final goal of building a control biosystem. As new data from forthcoming seasons becomes available, the predictive models will gain in precision and consistency, quantitatively determined by their statistical significance. Other crops will certainly require different input maps, and new traits or better assessment of current ones will eventually expand the set of input maps for vineyard production. The application of Model 15Y to the input map of Figure 7 resulted in the prediction yield of Figure 34, whereas the application of Model 3Q to the same input map led to the QPI predictions of Figure 35. Likewise, the application of Model 15Y§ to the input map of Figure 28 produced the low resolution prediction map of Figure 36, and Model 3Q§ on the same input map gave the QPI distribution of Figure 37. The benefits of improving the measurement of traits will be twofold; on one hand, input maps will be more truthful in representing the physical reality of the field; and on the other, predictive models will be more accurate in relating input and output maps. Regarding the prediction of yield, the measurements of Figure 9 can be compared to the predictions of Figure 34, and similarly for low resolution, Figure 29 should be compared to Figure 36. Overall, east-west trends coincide in predictions and measurements, but the former show a more uniform yield than the actual ones and smaller values in the east side. Quality predictions estimated by means of the QPI allow the comparison of measurements in Figure 25 with the predictions of Figure 35, and the corresponding contrast for low resolution between Figure 31 and Figure 37. Predicted quality tends to be higher (more cells with QPI ≈ 0) than measured quality but both predictions (low and high resolution) conserve the same lack of east-west spatial dominance that was found in the measurements of Figures 25 and 31.

## 7. Conclusions and Future Work

The methodology of Crop Biometric Maps establishes a general framework to manage PA-based IT-oriented field data. The proposed format for the CB maps fosters the compatibility among years, diverse technologies, and variables (traits) of any nature, which is essential for the universal dissemination of precision agriculture. The fact that users themselves can decide key features on field information, such as map resolution, coordinates of local origins, and crop traits, facilitates the seamless welding of emergent technologies with the reality of agricultural production, reducing the vast gap between theoretical academics and field implementation by average growers rather than IT experts. CB maps, in addition, allow the easy display of information supported by a strong mathematical background; users do not need to dive into the statistical generation of models, they just need to interpret the maps and make managerial decisions accordingly. The own nature of the system includes the capacity of improving the prediction models continuously as data can be permanently being input to the system. The framework envisioned along this paper has a broad spectrum as it represents one philosophy for many crops and production systems.

The particular application of CB maps to vineyards showed the advantages of the method and led to motivating conclusions. Important practices traditionally justified by conventional wisdom were quantified and statistically analyzed. In particular, grape yield was predicted from an automatic assessment of vegetation growth along ten rows of vines. A normalized quality index, the QPI, was defined, computed, and also correlated with the spatial variability of vine vigor. Soil properties did not contribute to the early estimation of yield, and neither yield nor berry size and density did affect the prediction of grape quality. The longstanding direct relation between vigor and yield, and quality drops for exuberant foliage and plentiful water were corroborated and numerically assessed in either low resolution or high resolution maps. The versatility of CB maps was demonstrated by arriving at the same conclusions for both map resolutions, and by proving that manual sampling and automatically acquired data all merge smoothly into standard maps.

Despite all the advantages found in CB maps, or perhaps because of them, the ultimate benefits of this method are still to come. In relation to vineyards and winemaking, progress will lead to the best measurement of traits, as most of the key parameters are already known to viticulturists. In particular, grape yield needs to be mapped from on-the-harvester yield monitors, and a better assessment of the spatial distribution of vine vigor is crucial to define more robust predictive models. All properties related to the berries, typically sugar content, acidity, or concentration of polyphenols, are currently measured invasively and manually. Any advance towards automating these estimations will definitely have a great impact on the modernization of vineyard management, and of course, will ease and enhance the construction of CB maps. The application of crop biometric maps to vineyards served as a concept-proof case study, but the ultimate objective is their generalized use in many other crops. Each crop will usually involve specific production requirements and that will steer the selection of particular traits for each case, but the ideal situation to be expected in the upcoming years would lead to the development of innovative biometric maps for key specialty crops such as citrus, persimmon, kiwi, nuts, cherries, olives, apples, and many more.

## Figures and Tables

**Table 1.** Selection of crop biometric traits for wine production vineyards.

	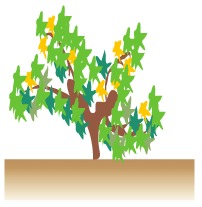	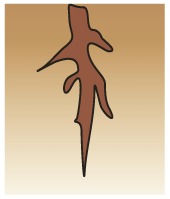	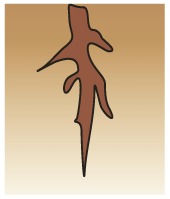	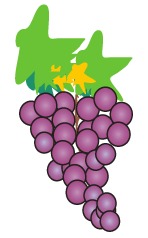	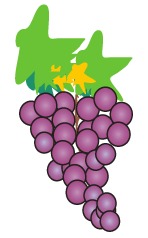	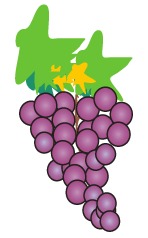	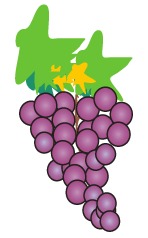	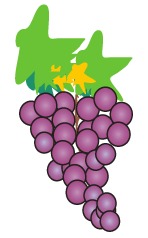

	**Vigor**	**Altitude**	**Soil Res.**	**Yield**	**Acidity**	**Sugar**	**Berry Size**	**pH**

**Cost**	Medium	Low	Medium	High	High	High	High	High
**Automation**	High	High	Low	Low	Low	Low	Low	Low
**Reliability**	Medium	High	Low	High	High	High	High	High
**Speed**	High	High	Medium	Low	Low	Low	Low	Low
**Correlation**	High	High	Low	High	High	Low	Low	High
**Interest**	High	Low	Low	High	High	High	Low	High
**Area samp.**	High	High	Low	H-L	Low	Low	Low	Low

**Table 2.** Inference statistics for vineyard biometric traits.

**Crop Parameter Tracked**	**n**	**Max**	**Min**	**Average**	**Median**	**St. Dev.**	**Normality**
Average soil resistance (MPa)	218	5	1.6	2.74	2.63	0.68	Strong
Maximum soil resistance (MPa)	218	8.5	2.3	4.8	4.5	1.4	Strong
Elevation (cm)	273	321	1	85.3	31	97.3	Weak
Vine vigor with 8 mm lens (%)	274	69	1	39.5	41	14.8	Strong
Vine vigor with 12 mm lens (%)	276	88	13	47.6	43	16.6	Strong
Grape yield (Kg/16 m^2^)	219	8.4	0.7	4.1	4	1.84	Very strong
Sugar content in juice (° Baumé)	219	15.1	10.1	13.1	13.2	0.62	Strong
Total acidity (g/L)	219	13	2.8	7.5	7.1	1.8	Very strong
Must pH	219	4.3	2.8	3.3	3.3	0.2	Very strong
Weight of 10 berries (g)	219	13.4	4.8	9.4	9.5	1.61	Very strong
Diameter of berries (mm)	219	12.9	8.9	11	11.1	0.74	Very strong
Berry density (g/cm^3^)	219	1.9	0.9	1.36	1.3	0.17	Strong

**Table 3.** Regression analysis between vegetation indices V-8 and V-12.

**Model**	***R****^2^*	$R_{\text{res}}^{2}$
*V*_8_ = 12.679 + 0.548 · *V*_12_	0.384	0.677
$V_{8}=-15.5477+1.7631\cdot V_{12}-0.0117\cdot V_{12}^{2}$	0.429	0.687
$V_{8}=-33.1902+2.9274\cdot V_{12}-0.0353\cdot V_{12}^{2}+0.0001\cdot V_{12}^{3}$	0.432	0.695

**Table 4.** Regression analysis between average and maximum soil resistance.

**Model**	***R****^2^*	$R_{\text{res}}^{2}$
*SR_max_* = 0.2773 + 1.6472 · *SR_av_SR_av_* > 1 *MPa*	0.632	0.695
$SR_{\max}=-1.1854+2.6863\cdot SR_{av}-0.1738\cdot SR_{av}^{2}\;SR_{av}>1\text{MPa}$	0.638	0.693

**Table 5.** Summary table for yield prediction models.

**Model**		***R****^2^*	$R_{\text{res}}^{2}$	**F-Stat**	**t-Stat Non-Signif.**
1Y	*Y* = 2.382 + 0.028 · *X*_1_ + 0.017 · *X*_2_ − 0.007 · *X*_3_ + 0.22 · *X*_4_ − 0.041 · *X*_5_	0.42	0.38	20.59	*X*_2_; *X*_4_; *X*_5_
2Y	*Y* = 3.146 + 0.034 · *X*_1_ − 0.008 · *X*_3_ + 0.093 · *X*_4_	0.41	0.42	33.01	*X*_4_
3Y	*Y* = 3.336 + 0.035 · *X*_1_ − 0.008 · *X*_3_	0.41	0.41	49.62	
4Y	*Y* = 1.228 + 0.07 · *X*_1_ + 0.046 · *X*_4_	0.34	0.46	37.86	*X*_4_
5Y	*Y* = 1.389 + 0.066 · *X*_1_ − 0.016 · *X*_4_ + 0.002 · *X*_1_ · *X*_4_	0.34	0.45	25.07	*X_4_*; *X*_1_ · *X*_4_
6Y	*Y* = 3.364 + 0.035 · *X*_1_ − 0.008 · *X*_3_ − 1.1569 · 10^−6^ · *X*_1_ · *X*_3_	0.41	0.41	32.85	*X*_1_ · *X*_3_†
7Y	*Y* = 0.204 + 0.072 · *X*_1_ + 0.037 · *X*_2_ − 3.5294 · 10^−4^ · *X*_1_ · *X*_2_	0.37	0.37	28.15	Constant; *X*_2_; *X*_1_; *X*_2_
8Y	*Y* = 1.346 + 0.07 · *X*_1_	0.34	0.47	76.15	
9Y	*Y* = 5.063 − 0.013 · *X*_3_	0.37	0.41	86.34	
10Y	*Y* = 1.423 − 0.057 · *X*_2_	0.22	0.23	42.16	
11Y	$Y=0.3676+0.1367\cdot X_{1}-0.0009\cdot X_{1}^{2}$	0.36	0.49	40.61	Constant; $X_{1}^{2}$
12Y	$Y=5.4519-0.0281\cdot X_{3}+0.0001\cdot X_{3}^{2}$	0.41	0.36	50.58	
13Y	$Y=1.359-0.0053\cdot X_{1}+0.0041\cdot X_{1}^{2}-4.96\cdot 10^{-5}\cdot X_{1}^{3}$	0.37	0.49	80.15	
14Y	*Y* = −1.5745 + 1.5970 · ln *X*_1_	0.3	0.43	62.60	
15Y	$Y=0.5724\cdot X_{1}^{0.5212}$	0.37	0.50	87.55	
16Y	$Y=4.3144-\frac{5.9086}{X_{1}}$	0.09	0.08	14.81	
17Y	*Y* = 3.975 − 1.242 · *Z*_3_	0.37	0.41	86.34	
18Y	*Y* = 2.67 + 0.034 · *X*_1_ − 0.791 · *Z*_3_ − 1.126 · 10^−4^ · *X*_1_ · *Z*_3_	0.41	0.41	32.85	*X*_1_ · *Z*_3_†
19Y	*Y* = 2.67 + 0.035 · *X*_1_ − 0.794 · *Z*_3_	0.41	0.41	49.62	

† Possible collinearity.

**Table 6.** ANOVA results for Model 1Y of Table 5.

***Y*** = **2.382** + **0.28** · ***X*_1_** + **0.017** · ***X*_2_** − **0.007** · ***X*_3_** + **0.22** · ***X*_4_** − **0.041** · ***X*_5_**					
*R*^2^	0.420				
$R_{\text{res}}^{2}$	0.379				
Model	1 Y				

Regression ANOVA					

Source	Df	Sum Sq	MeanSq	F-stat	*P*-value
Regression	5.00	202.8338	40.5668	20.5863	0.00
Error	142.00	279.8210	1.9706		
Total	147.00	482.6548			

t-Statistics for Predictor Variables					

Variable		Estimate	St. error	*t*-value	*P*-value
Constant		2.3820	0.8547	2.7869	0.0060
*X*_1_		0.0277	0.0125	2.2048	0.0291
*X*_2_		0.0171	0.0099	1.7248	0.0867
*X*_3_		−0.0073	0.0021	−3.4388	0.0008
*X*_4_		0.2197	0.2909	0.7553	0.4513
*X*_5_		−0.0410	0.1351	−0.3038	0.7617

**Table 7.** ANOVA results for Model 5Y of Table 5.

***Y*** = **1.389** + **0.066** · ***X*_1_** − **0.016** · ***X*_4_** + **0.002** ·***X*_1_** · ***X*_4_**					
*R*^2^	0.343				
$R_{\text{res}}^{2}$	0.455				
Model	5 Y				

Regression ANOVA					

Source	Df	Sum Sq	MeanSq	F-stat	*P*-value
Regression	3.00	165.6109	55.2036	25.0733	0.00
Error	144.00	317.0439	2.2017		
Total	147.00	482.6548			

t-Statistics for Predictor Variables					

Variable		Estimate	St. error	*t*-value	*P*-value
Constant		1.3889	1.4688	0.9456	0.3459
*X*_1_		0.0661	0.0344	1.9189	0.0570
*X*_4_		−0.0155	0.5482	−0.0283	0.9775
*X*_1_ · *X*_4_		0.0015	0.0127	0.1196	0.9050

**Table 8.** ANOVA results for Model 15Y of Table 5.

$Y=0.5724\cdot X_{1}^{0.5212}$					
R^2^	0.375				
$R_{\text{res}}^{2}$	0.503				
Model	15 Y				

Regression ANOVA					

Source	Df	Sum Sq	MeanSq	F-stat	*P*-value
Regression	1.00	15.4286	15.4286	87.5534	0.00
Error	146.00	25.7281	0.1762		
Total	147.00	41.1567			

t-statistics for predictor variables					

Variable		Estimate	St. error	*t*-value	*P*-value
Constant		−0.5580	0.1995	−2.7964	0.0059
*X*_1_		0.5212	0.0557	9.3570	0.0000

**Table 9.** Summary table for QPI prediction models.

**Model**		***R****^2^*	$R_{\text{res}}^{2}$	**F-Stat**	**t-Stat Non-Signif.**
1Q	*QPI* = −0.846 − 0.006 · *X*_1_ − 0.057 · *X*_11_ + 0.122 · *X*_12_ + 0.216 · *X*_13_ + 0.002 · *X_y_*	0.32	0.23	18.13	Constant; *X*_11_; *X*_12_; *X*_13_; *X_y_*
2Q	*QPI* = 0.407 − 0.006 · *X*_1_ − 0.096 · *X*_13_ + 0.002 · *X_y_*	0.31	0.17	29.31	*X*_13_; *X_y_*
3Q	***QPI*** = **0.289** − **0.007** · ***X*_1_**	0.31	0.24	85.33	
4Q	*QPI* = 0.331 − 0.221 · *X*_13_	0.05	0.17	9.34	
5Q	*QPI* = 0.146 − 0.028 · *X*_y_	0.09	0.01	18.7	
6Q	*QPI* = 0.372 − 0.036 · *X*_11_	0.11	0.16	24.02	
7Q	*QPI* = 0.434 − 0.037 · *X*_12_	0.02	0.06	4.54	
8Q	*QPI* = 0.338 − 0.008 · *X*_1_ − 0.015 · *X_y_* + 4.2 · 10^−4^ · *X*_1_ · *X_y_*	0.31	0.29	28.64	*X_y_*; *X*_1_; *X_y_*
9Q	*QPI* = 0.286 − 0.007 · *X*_1_ + 0.002 · *X_y_*	0.31	0.29	42.52	*X_y_*
10Q	$\text{QPI}=0.2561-0.0044\cdot X_{1}-0.00003\cdot X_{1}^{2}$	0.31	0.32	42.78	*X*_1_; $X_{1}^{2}$
11Q	*QPI* = 0.5451 − 0.1444 · ln *X*_1_	0.24	0.12	60.06	
12Q	$\text{QPI}=-0.2562\cdot X_{1}^{-0.3365}$	0.27	0.01	25.61	
13Q	$\text{QPI}=0.0101+\frac{0.5248}{X_{1}}$	0.06	0 *	12.83	Constant

* Negative value.

**Table 10.** Summary table for yield prediction models adapted to low resolution maps.

**Low Resolution Model**		***R****^2^*	$R_{\text{res}}^{2}$	**F-Stat**	**T-Stat Non-Signif.**
1Y§	*Y* = 8.404 − 0.028 · *X*_1_ + 0.012 · *X*_2_ − 0.007 · *X*_3_ + 1.227 · *X*_4_ − 1.328 · *X*_5_	0.53	0.60	12.9	*X*_1_; *X*_2_
2Y§	*Y* = 6.392 + 0.007 · *X*_1_ − 0.009 · *X*_3_ − 0.641 · *X*_4_	0.37	0.66	11.4	*X*_1_; *X*_4_
3Y§	*Y* = 4.145 + 0.019 · *X*_1_ − 0.009 · *X*_3_	0.33	0.55	15.0	*X*_1_
4Y§	*Y* = 4.317 + 0.045 · − *X*_1_ − 0.009 · *X*_4_	0.28	0.49	11.7	*X*_4_
5Y§	*Y* = 4.546 + 0.039 · *X*_1_ − 0.755 · *X*_4_ + 0.002 · *X*_1_ · *X*_4_	0.28	0.48	7.6	Constant; *X*_1_; *X*_4_; *X*_1_ · *X*_4_
6Y§	*Y* = 4.143 + 0.019 · *X*_1_ − 0.009 · *X*_3_ − 5.46 · 10^−7^ · *X*_1_ · *X*_3_	0.33	0.54	9.8	*X*_1_; *X*_3_; *X*_1_ · *X*_3_^†^
7Y§	*Y* = 0.917 + 0.066 · *X*_1_ + 0.026 · *X*_2_ − 2.55 · 10^−4^ · *X*_1_ · *X*_2_	0.26	0.62	6.9	All
8Y§	*Y* = 1.88 + 0.059 · *X*_1_	0.24	0.55	19.4	
9Y§	*Y* = 5.093 − 0.011 · *X*_3_	0.32	0.54	28.9^†^	
10Y§	*Y* = 2.786 + 0.033 · *X*_2_	0.08	0.5	5.6	
11Y§	$Y=1.1457+0.1060\cdot X_{1}-0.0006\cdot X_{1}^{2}$	0.25	0.56	10.1	Constant; $X_{1}^{2}$
12Y§	$Y=5.3189-0.0206\cdot X_{3}+3.53\cdot 10^{-5}\cdot X_{3}^{2}$	0.34	0.59	15.2	$X_{3}^{2}$
13Y§	$Y=1.7993-0.0048\cdot X_{1}+0.0033\cdot X_{1}^{2}-3.91\cdot 10^{-5}\cdot X_{1}^{3}$	0.27	0.67	7.2	
14Y§	*Y* = 0.1393 + 1.152 · ln *X*_1_	0.21	0.41	16	Constant
15Y§	$Y=1.0623\cdot X_{1}^{0.3626}$	0.3	0.5	26.3	Constant
16Y§	$Y=4.4538-\frac{4.0956}{X_{1}}$	0.10	0.17	7	
17Y§	*Y* = 4.119 − 1.111 · *Z*_3_	0.32	0.54	28.9^†^	
18Y§	*Y* = 3.374 + 0.019 · *X*_1_ − 0.878 · *Z*_3_ − 5.31 · 10^−5^ · *X*_1_ · *Z*_3_	0.33	0.54	9.8	*X*_1_; *Z*_1_; *X*_1_ · *Z*_3_^†^
19Y§	*Y* = 3.374 + 0.019 · *X*_1_ − 0.879 · *Z*_3_	0.33	0.55	15	*X*_1_^†^

† Possible collinearity.

**Table 11.** Summary table for QPI prediction models adapted to low resolution maps.

**Low Resolution Model**		***R****^2^*	$R_{\text{res}}^{2}$	**F-Stat**	**T-Stat Non-Signif.**
1Q§	*QPI* = −1.96 − 0.005 · *X*_1_ − 0.123 · *X*_11_ + 0.249 ·*X*_12_ + 0.437 · *X*_13_ − 0.001 · *X_y_*	0.48	0.44	11.4	Constant; *X*_12_; *X*_13_; *X_y_*
2Q§	*QPI* = 0.403 − 0.006 · *X*_1_ − 0.105 · *X*_13_ − 0.002 · *X_y_*	0.42	0.39	15.3	*X*_13_; *X_y_*
3Q§	***QPI*** = **0.265** − **0.0062** · ***X*_1_**	0.41	0.39	45.9	
4Q§	*QPI* = 0.041 − 0.292 · *X*_13_	0.05	0.02	3.6	Constant; *X*_13_
5Q§	*QPI* = 0.133 − 0.027 · *X_y_*	0.11	0*	8.2	
6Q§	*QPI* = 0.473 − 0.049 · *X*_11_	0.26	0.13	23.3	
7Q§	*QPI* = 0.916 − 0.081 · *X*_12_	0.14	0 *	11	
8Q§	*QPI* = 0.338 − 0.008 · *X*_1_ − 0.024 · *X_y_* + 10^−3^ · *X*_1_ · *X_y_*	0.42	0.41	15.3	*X_y_*; *X*_1_ · *X_y_*
9Q§	*QPI* = 0.269 − 0.006 · *X*_1_ − 0.002 · *X_y_*	0.41	0.40	22.6	*X_y_*
10Q§	$\text{QPI}=0.197-0.0016\cdot X_{1}-0.000062\cdot X_{1}^{2}$	0.42	0.51	24	$X_{1};X_{1}^{2}$
11Q§	*QPI* = 0.4144 − 0.1112 · ln *X*_1_	0.29	0.13	26.5	
12Q§	$\text{QPI}=0.0888\cdot X_{1}^{-0.2759}$	0.12	0.2	6.1	Constant
13Q§	$\text{QPI}=4.7\cdot 10^{-5}+\frac{0.3864}{X_{1}}$	0.13	0.1	9.6	Constant

* Negative value.
